# Signature proteins for the major clades of Cyanobacteria

**DOI:** 10.1186/1471-2148-10-24

**Published:** 2010-01-25

**Authors:** Radhey S Gupta, Divya W Mathews

**Affiliations:** 1 Department of Biochemistry and Biomedical Sciences, McMaster University, Hamilton, Ontario, Canada L8N 3Z5

## Abstract

**Background:**

The phylogeny and taxonomy of cyanobacteria is currently poorly understood due to paucity of reliable markers for identification and circumscription of its major clades.

**Results:**

A combination of phylogenomic and protein signature based approaches was used to characterize the major clades of cyanobacteria. Phylogenetic trees were constructed for 44 cyanobacteria based on 44 conserved proteins. In parallel, Blastp searches were carried out on each ORF in the genomes of *Synechococcus WH8102, Synechocystis PCC6803, Nostoc PCC7120, Synechococcus JA-3-3Ab, Prochlorococcus MIT9215 *and *Prochlor. marinus subsp. marinus CCMP1375 *to identify proteins that are specific for various main clades of cyanobacteria. These studies have identified 39 proteins that are specific for all (or most) cyanobacteria and large numbers of proteins for other cyanobacterial clades. The identified signature proteins include: (i) 14 proteins for a deep branching clade (Clade A) of *Gloebacter violaceus *and two diazotrophic *Synechococcus *strains (JA-3-3Ab and JA2-3-B'a); (ii) 5 proteins that are present in all other cyanobacteria except those from Clade A; (iii) 60 proteins that are specific for a clade (Clade C) consisting of various marine unicellular cyanobacteria (viz. *Synechococcus *and *Prochlorococcus*); (iv) 14 and 19 signature proteins that are specific for the Clade C *Synechococcus *and *Prochlorococcus *strains, respectively; (v) 67 proteins that are specific for the Low B/A ecotype *Prochlorococcus *strains, containing lower ratio of *chl b/a*_2 _and adapted to growth at high light intensities; (vi) 65 and 8 proteins that are specific for the *Nostocales *and *Chroococcales *orders, respectively; and (vii) 22 and 9 proteins that are uniquely shared by various *Nostocales *and *Oscillatoriales *orders, or by these two orders and the *Chroococcales*, respectively. We also describe 3 conserved indels in flavoprotein, heme oxygenase and protochlorophyllide oxidoreductase proteins that are specific for either Clade C cyanobacteria or for various subclades of *Prochlorococcus*. Many other conserved indels for cyanobacterial clades have been described recently.

**Conclusions:**

These signature proteins and indels provide novel means for circumscription of various cyanobacterial clades in clear molecular terms. Their functional studies should lead to discovery of novel properties that are unique to these groups of cyanobacteria.

## Background

Cyanobacteria are the sole prokaryotic group that carries out oxygenic photosynthesis. The species from this phylum exhibit enormous diversity in terms of their morphology, physiology and other characteristics (e.g. motility, thermophily, cell division characteristic, nitrogen fixation ability, etc.) [1-5]. The taxonomy and evolutionary relationships among cyanobacteria is presently poorly understood. In the 16S rRNA trees, which provides the current basis for understanding microbial phylogeny, cyanobacteria species/strains form 14 unresolved clusters [6]. Although cyanobacteria is a large phylum with >4000 isolates [7], only a small number of species and higher taxonomic groups within this phylum have been validly described [8-10]. Except for 16S rRNA, sequence information for cyanobacteria for other genes/proteins sequences until recently was very limited. Hence, the availability of genome sequences has provided new opportunities for understanding cyanobacterial phylogeny and taxonomy. Based upon these sequences, several investigators have assembled phylogenetic trees for cyanobacteria based upon combined sequences for different large sets of proteins. These studies have included analyses of 14 cyanobacteria based upon 34 proteins by Sanchez-Barcaldo et al. [4], trees for 24 cyanobacteria based upon 583 orthologous proteins by Swingley et al. [11], and branching patterns of 13 cyanobacteria based upon 682 proteins by Shi and Falkowski [12]. Additionally, Zhaxybayeva et al. [13] have examined individual phylogenies of 1128 protein-coding genes from 11 cyanobacterial genomes to identify phylogenetic signal exhibited by the plurality of these proteins and to recognize the incidence of lateral gene transfers. These studies have proven very useful in establishing the existence of certain important clades within the sequenced cyanobacteria and in clarifying their relative branching positions [4,11,12].

The studies of the above kind, although very useful, are limited to species whose genomes are sequenced. Further, as indicated by earlier work [4,11,12], integration of sequence information from any new genome by this approach requires reassembly of the entire phylogenomic tree(s). Based upon the phylogenomic approach it is also difficult to circumscribe various cyanobacterial clades in definitive biochemical or molecular terms, which is important for developing a stable taxonomy [14-16]. Hence, it is important to identify other reliable molecular markers that are consistent with the results of phylogenomic studies, but which can also be used to circumscribe different phylogenetic clades in more definitive (molecular) terms. One approach that has proven very useful in this regard consists of identifying molecular markers or synapomorphies that are specific for different phylogenetically defined clades. Two different kinds of molecular markers are proving very useful for these studies. The first of these consists of conserved inserts and deletions (indels) in widely distributed proteins that are distinctive characteristics of either a given phylum or its different main subgroups [17-21]. Our recent work has identified >40 conserved indels in important proteins that are exclusively present in either all cyanobacteria or many of its major clades that are observed in phylogenomic trees [22,23]. The presence of several of these indels in the plants/plastids homologs has also provided evidence for the derivation of plastids from cyanobacterial ancestors [22-24]. The second kind of molecular markers consists of whole proteins that are uniquely found in various species from a given phylogenetic clade [25-28]. Martin et al. [29] have earlier reported Blast analysis on 8 cyanobacterial genomes (6 finished and 2 unfinished) to identify 181 proteins that were uniquely found in at least 7 out of 8 of these cyanobacteria. A later study by Mulkidjanian et al. [30] on 15 cyanobacterial genomes identified 50 proteins that were uniquely present in at least 14 out of 15 cyanobacteria and 84 others that were exclusively present in plants/plastids and cyanobacteria.

These earlier studies primarily looked for proteins that were uniquely found in most cyanobacteria and no work was carried out on identifying proteins that are specific for various main clades of cyanobacteria, observed in phylogenetic trees. In the past 2-3 years, the number of sequenced cyanobacterial genomes has also more than doubled to a total of 36 genomes. Hence, it was of much interest to carry out both phylogenomic as well as gene content analyses on these genomes to identify signature proteins that are distinctive characteristics of either all cyanobacteria or its various main clades in the phylogenomic trees.

## Results

### Phylogenomic/phylogenetic analyses on Cyanobacteria

Prior to undertaking studies on identifying proteins that are specific for different cyanobacterial clades, it was necessary to determine the branching pattern of sequenced cyanobacteria in phylogenetic trees. Although detailed phylogenetic studies have been previously reported for a limited numbers of cyanobacteria [4,11,12], sequence information for many other genomes has become available in the past 2-3 years (see Table 1). Hence, it was necessary to carry out phylogenetic studies on all of these cyanobacteria to determine their branching pattern. The phylogenetic trees are now commonly constructed based on concatenated sequences for large number of proteins [4,11,12,31]. Their main advantage is that because they are based on large numbers of characters derived from many independent proteins, they are generally considered to provide a better reflection of organismal phylogeny than trees based on any single gene or protein, where the observed relationship could be affected by various factors including lateral gene transfer, differences in evolutionary rates among species, long branch attraction effect, etc. [32]. However, it should be recognized that the trees based on concatenated sequences, due to the possibility of their lumping together gene sequences with discordant evolutionary histories, can sometime result in unreliable inferences [32-34]. In the present work, phylogenetic trees were constructed based on a combined sequence alignment for 44 widely distributed proteins (see additional file 1) from 44 cyanobacterial species/isolates for which sequence information was available (see Materials and Methods). Most of these proteins carry out important housekeeping functions, and they are universally present in various species [35], making them a good choice for phylogenetic analysis.

**Table 1:** List of Cyanobacterial Genomes Studied in this work

Species Name	Genome size (Mb)	GC content %	Protein Number	Genome Reference	Center/Pubmed ID
*Acaryochloris marina MBIC11017*	8.36	47.0	6254	NC_009925.1	[45]
*Anabaena variabilis ATCC 29413*	7.07	41.4	5043	NC_007413.1	DOE JGI
*Gloeobacter violaceus PCC 7421*	4.66	62	4430	NC_005125.1	[36]
*Cyanothece sp. ATCC 51142*	5.43	37.9	4762	NC_010546.1	Washington University
*Cyanothece sp. PCC 8801*	4.81	39.8	4260	NC_011726.1	DOE JGI
*Nostoc sp. PCC 7120*	7.21	41.3	5366	NC_003272.1	[48]
*Microcystis aeruginosa NIES-843*	5.8	42.3	6312	NC_010296.1	Kazusa
*Nostoc punctiforme PCC73102*	8.2	41.4	6087	NC_010628.1	DOE JGI
*Prochloro. marinus str. AS9601*	1.7	31.3	1921	NC_008816.1	J. Craig Venter Institute
*Prochloro. marinus str. MIT 9211*	1.7	39.7	1855	NC_009976.1	[40]
*Prochloro. marinus str. MIT 9215*	1.7	31.1	1983	NC_009840.1	DOE JGI
*Prochloro. marinus str. MIT 9301*	1.6	31.3	1907	NC_009091.1	GBM Foundation
*Prochloro. marinus str. MIT 9303*	2.7	50	2997	NC_008820.1	J. Craig Venter Institute
*Prochloro. marinus str. MIT 9312*	1.71	31.2	1810	NC_007577.1	DOE JGI.
*Prochloro. marinus str. MIT 9313*	2.41	50.7	2269	NC_005071.1	[40]
*Prochloro. marinus str. MIT 9515*	1.7	30.8	1906	NC_008817.1	J. Craig Venter Institute
*Prochloro. marinus str. NATL1A*	1.9	35	2193	NC_008819.1	J. Craig Venter Institute
*Prochloro. marinus str. NATL2A*	1.8	35.1	2163	NC_007335.2	DOE Joint Genome Inst.
*Prochloro. marinus subsp. marinus str. CCMP1375*	1.75	36.4	1883	NC_005042.1	[51]
*Prochloro. marinus subsp. pastoris str. CCMP1986*	1.7	30.8	1717	NC_005072.1	[40]
*Synechococcus elongatus PCC 6301*	2.7	55.5	2527	NC_006576.1	[83]
*Synechococcus elongatus PCC 7942*	2.75	55.4	2612	NC_007604.1	DOE JGI
*Synechococcus sp. CC9311*	2.61	52.4	2892	NC_008319.1	[53]
*Synechococcus sp. CC9605*	2.51	59.2	2645	NC_007516.1	[84]
*Synechococcus sp. CC9902*	2.23	54.2	2307	NC_007513.1	[84]
*Synechococcus sp. JA-2-3B'a(2-13)*	3.05	58.5	2862	NC_007776.1	TIGR
*Synechococcus sp. JA-3-3Ab*	2.93	60.2	2760	NC_007775.1	TIGR
*Synechococcus sp. RCC307*	2.2	60.8	2535	NC_009482.1	[84]
*Synechococcus sp. WH7803*	2.4	60.2	2533	NC_009481.1	[84]
*Synechococcus sp. PCC 7002*	3.4	49.2	2823	NC_010475.1	Penn. State University
*Synechococcus sp. WH8102*	2.43	59.4	2519	NC_005070.1	[52]
*Synechocystis sp. PCC 6803*	3.95	47.4	3172	NC_000911.1	[85]
*Thermosynechococcus elongatus BP-1*	2.59	53.9	2476	NC_004113.1	[46]
*Trichodesmium erythraeum IMS101*	7.8	34.1	4451	NC_008312.1	DOE Joint Genome Inst.

Abbreviations: DOE-JGI, Department of Energy Joint Genome Institute; TIGR, The Institute of Genome Research; GBM, Gordon & Betty Moore. The genome of *Crocosphaera watsonii *WH8501 was not fully sequenced.

**Figure 1:**
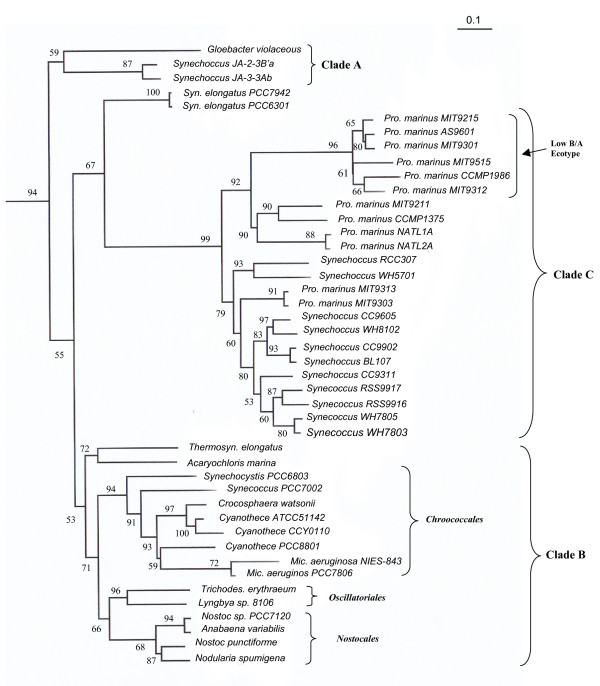
**A maximum-likelihood distance tree for sequenced cyanobacteria based on concatenated sequences for 44 conserved proteins**. The distance scale (bar = 0.1 substitutions per site) is shown in the top right hand corner. The tree was rooted using *B. subtilis *and *S. aureus *sequences. The numbers at the nodes indicate % of puzzling quartets supporting various nodes. The low B/A ecotype clade refers to the *Prochlorococcus *spp. containing lower ratio of chlorophyll **b/a_2 _**that are adapted to growth at high light intensities.

A rooted maximum likelihood (ML) distance tree based on the combined sequences for these proteins is shown in Fig. 1 and a neighbour-joining (NJ) tree for the same dataset is provided as additional file 2. A number of distinct clades of cyanobacteria were observed in both these trees. Very similar branching patterns and the grouping of cyanobacterial species in various clades have been observed in earlier studies based on other large and independent datasets of protein sequences [4,11,12], giving confidence in the observed results. One of the observed clades, referred to here as Clade A, consists of *Gloebacter violaceus *and *Synechococcus sps*. (JA-3-3Ab and JA2-3-B'a). The ML and NJ tree differ from each other in the branching position of this clade. In the ML tree, the Clade A species/strains formed the deepest branching lineage within cyanobacteria. In contrast, in the NJ tree, the cyanobacteria were divided into two main clades at the deepest level and the Clade A formed the outermost branch of one of these clades, separated from all other species/strains by a long branch (additional file 2). However, the branching of Clade A in this position is not reliable, as in our recent studies based on the same dataset of protein sequences but with smaller numbers of cyanobacteria, the clade A species/strains branched in the same position as seen here in the ML tree [23]. The deep branching of Clade A species/strains has also been observed in a number of earlier studies based on different datasets of protein sequences [4,6,11,12,23,36-39]. Further strong and independent evidence that the Clade A species/strains constitutes the earliest branching lineage within sequenced cyanobacteria is provided by our recent identification of several conserved indels in broadly distributed proteins (viz. 18 aa insert in DNA polymerase I, 4-5 aa insert in the tryptophan synthase beta chain, 4 aa insert in tryptophanyl-tRNA synthetase and a 2 aa insert in the DNA polymerase III) [23]. The indicated conserved inserts in these proteins are commonly shared by all other sequenced cyanobacteria, but they are lacking in Clade A as well as all other phyla of bacteria [23]. The species distributions of these conserved indels strongly indicate that these synapomorphies were introduced in a common ancestor of various other cyanobacteria after the branching of Clade A. In a recent proposal for the classification of cyanobacteria, the thylakoids lacking *Gloebacterales *are placed into a separate subclass (Gloebacterophycidae) [15]. It is unclear whether the *Synechococcus sps*. (JA-3-3Ab and JA2-3-B'a), which group with *G. violaceus*, also lack thylakoids or not.

Most other cyanobacteria could be grouped into two main clades in these trees. One of these clades (designated here as Clade B) is comprised of diverse cyanobacteria including *Thermosynechococcus, Acaryochloris*, as well as other cyanobacterial groups such as *Chroococcales *(*Synechocystis/Crocosphaera/Microcystis/Cyanothece*), *Nostocales *(*Nostoc/Nodularia/Anabaena*) and *Oscillatoriales *(*Trichodesmium/Lynbya*)[15]. Within Clade B, a subclade comprising of the *Chroococcales, Nostocales *and *Oscillatoriales *is also observed in both ML and NJ trees (Fig. 1 and additional file 2). The other main clade (clade C) is composed entirely of different strains/isolates of marine unicellular *Prochlorococcus *and *Synechococcus *cyanobacteria. This latter clade has been referred to as the Syn/Pro clade [4] and it corresponds to the subclass Synechococcophycidae in the proposal by Hoffman et al. [15]. Within clade C, different *Prochlorococcus *and *Synechococcus *strains/isolates were not completely separated from each other. In particular, two of the *Prochlorococcus *strains, MIT 9303 and MIT 9313, branched within the *Synechoccous *strains/isolates, in both ML and NJ trees (Fig. 1 and additional file 2). Similar polyphyletic branching of these strains has been observed in earlier studies [12,23]. However, in both these trees, one subclade of *Prochlorococcus *strains, which is referred to as the low B/A ecotype subgroup [40,41], was separated from all others *Prochlorococcus *strains by a long-branch. The branching position of the freshwater unicellular cyanobacterium *Synechococcus elongatus *(strains PCC 6301 and PCC 7942), although it appeared as a deep branching lineage of Clade C, was uncertain in these trees (discussed later).

### Signature proteins for Cyanobacteria and its major subgroups

These phylogenetic trees provide a framework for identifying proteins that are specific for either all cyanobacteria or their different well-resolved clades. Based upon earlier studies, within any given group of bacteria or organisms, signature proteins are present at various phylogenetic depths [25,27,28,42-44]. Hence, to identify proteins that are specific for different main clades of cyanobacteria, Blastp searches were carried out on each ORF in the genomes of the following 6 cyanobacteria: *Synechococcus sp. WH8102, Synechocystis sp. PCC6803, Nostoc sp. PCC7120, Synechococcus sp. JA-3-3Ab, Prochlorococcus sp. MIT9215 *and *Pro. marinus subsp. marinus str. CCMP1375*. These cyanobacteria are present at the tips of various clades in phylogenetic trees (Fig. 1 and additional file 2). Hence, blast searches with the proteins in them should enable us to identify proteins that are specific for various main clades of cyanobacteria at different phylogenetic depths. The results of these studies are summarized below.

### Signature proteins that are specific for Cyanobacteria

Blast searches on the above genomes have identified 39 proteins that are specific for cyanobacteria and which are present in virtually all of the sequenced genomes (Table 2a). Thirty-three of these proteins are present in all sequenced cyanobacteria (Table 2a) whereas the remaining 6 (marked with *) are missing in 1-2 isolated species/strains. The homologs of some of these proteins are also found in a few algae or plants. Because of their specific presence in practically all cyanobacteria, but generally no other bacteria, these proteins could be regarded as the cyanobacterial signature proteins. The number of cyanobacterial signature proteins identified in the present work is much smaller than those reported in earlier studies [29,30]. However, this difference is mainly due to the large increase in the number of sequenced cyanobacterial as well as other genomes in the past few years. In earlier work, we have also described 15 conserved indels in broadly distributed proteins that are distinctive characteristics of all available cyanobacteria and which are not found in any other bacterial groups/phyla [22,23].

**Table 2:** Cyanobacterial Signature Proteins

(a) Protein that are Uniquely found in All (or most) Cyanobacteria			
**Protein**	**Function (length)**	**Protein**	**Function (length)**

NP_439901/slr0613	hypothetical (173)	NP_441893/ssl0242	hypothetical (78)

NP_439967/slr1122	hypothetical (329)	NP_442014/sll0350*	hypothetical (803)

NP_439995/slr0729^+^	hypothetical (101)	NP_442026/slr0376	hypothetical (116)

NP_440139/slr1796	hypothetical (201)	NP_442147/sll0208*	hypothetical (231)

NP_440262/ssl1972	hypothetical (93)	NP_442176/sll0413*	hypothetical (207)

NP_440437/slr2049^+^	hypothetical (192)	NP_442207/ssr0109	hypothetical (78)

NP_440459/slr1915	hypothetical (104)	NP_442330/sll0372 ^a^	hypothetical (196)

NP_440545/ssr2843^+^	hypothetical (87)	NP_442365/ssr0332	hypothetical (70)

NP_440678/slr1900 ^a^	hypothetical (247)	NP_442366/slr0211	hypothetical (403)

NP_440903/sll1271	hypothetical (572)	NP_442402/slr0921	hypothetical (128)

NP_440946/sll0860	hypothetical (173)	NP_442464/sll0822^a^	hypothetical (129)

NP_441021/ssr3189	hypothetical (55)	NP_442734/slr0042	hypothetical (576)

NP_441047/slr2144*	hypothetical (301)	NP_442826/sll1340	hypothetical (85)

NP_441164/ssr2087	hypothetical (84)	NP_442884/slr1557	hypothetical (369)

NP_441199/slr1990	hypothetical (240)	NP_442932/slr0748^+^	hypothetical (230)

NP_441265/ssl0461*	hypothetical (83)	NP_443015/sll1109	hypothetical (194)

NP_441307/sll1979	hypothetical (142)	NP_484529/asr0485^+^	hypothetical (92)

NP_441346/ssr2551	hypothetical (94)	NP_440513/slr1384	hypothetical (391)

NP_441647/slr1160*	hypothetical (204)	NP_0010358/slr1146	hypothetical (89)

NP_441848/sll0359	hypothetical (155)		

**(b) Proteins Specific for Various Cyanobacteria Except those from Clade A**			

NP_439997/slr0731	Hypothetical (402)	NP_441174/slr1260	Hypothetical (177)

NP_440149/slr1800	Hypothetical (355)	NP_441937/slr1949	Hypothetical (212)

NP_441115/sll0854	Hypothetical (308)		

**(c) Proteins Specific for Various Cyanobacteria Except those from Clade C**			

NP_440495/sll0984	Hypothetical (148)	NP_441597/slr1276	Hypothetical (275)

NP_440591/slr2025	Hypothetical (153)	NP_485360/all1317	Hypothetical (147)

NP_440594/sll1915	Hypothetical (183)	NP_488024/all3984	Hypothetical (231)

NP_440896/sll1274	Hypothetical (171)	NP_488046/all4006	Hypothetical (127)

NP_441155/sll1155*	Hypothetical (113)	NP_484683/asl0639	Hypothetical (73)

NP_484163/all0119*	Hypothetical (137)	NP_485187/alr1144*	Hypothetical (290)

NP_484255/all0211*	Hypothetical (126)		

* - missing in 1-2 species

^a ^significant similarity also seen for 1-2 other bacteria

^+ ^also found in some algae and mosses

Clade A is comprised of *G. violaceus, Synechococcus sp. JA-3-3Ab and Synechococcus sp. JA-2-3B'a*

Clade C is comprised of most of the *Synechococcus *and all *Prochlorococcus sps*.

**Figure 2:**
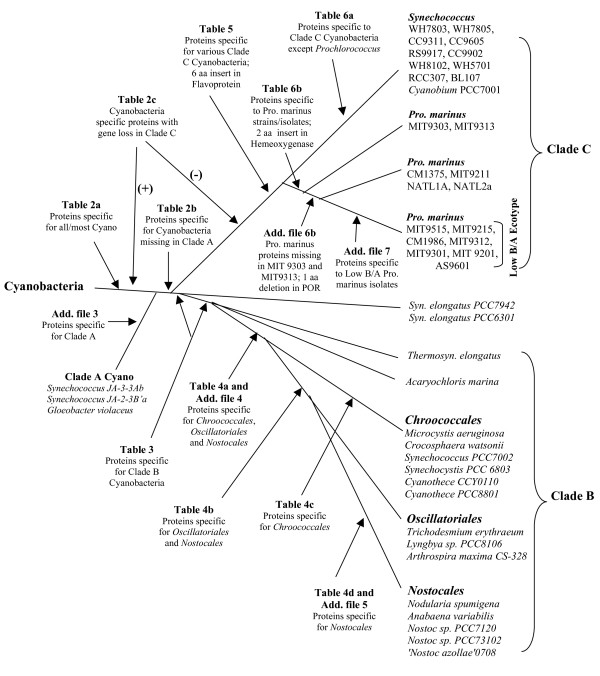
**An interpretive cladogram indicating the evolutionary stages where genes for different signature proteins described in this work, which are specific for different groups of cyanobacteria, likely evolved**. Many conserved indels that are specific for the same groups/clades of cyanobacteria, have also been described in recent work [23].

These analyses have also identified 5 proteins whose homologs are present in all other cyanobacteria, except those from Clade A (Table 2b). Based upon solely the genomic distributions of these proteins, it is difficult to interpret whether the genes for these proteins first evolved in a common ancestor of all cyanobacteria followed by their loss in Clade A species/strains, or they originally evolved in a common ancestor of the Clade B and C cyanobacteria after the branching of Clade A. However, based upon the results of phylogenomic analyses, and more importantly the species distribution patterns of several conserved indels in widely distributed proteins that provide evidence that the Clade A is ancestral to other cyanobacteria [23], the most parsimonious explanation for the observed distribution of these genes is that they first evolved in a common ancestor of the Clade B and C cyanobacteria, as indicated in Fig. 2. Table 2c lists 13 other proteins for which high scoring homologs are present in all (or most) cyanobacteria from Clades A and B, but which are lacking in Clade C strains/isolates. Because of the deep branching of Clade A, it is likely that the genes for these proteins also first evolved in a common ancestor of cyanobacteria, followed by their loss in an ancestor of Clade C. The alternate possibility that the Clade A and B cyanobacteria shared a common ancestor exclusive of Clade C is not supported by the species distribution pattern of conserved indels in several proteins, as noted above. Blast searches with proteins in the genome of *Synechococcus sp. JA-3-3Ab *have also identified 14 proteins that are specific for the Clade A cyanobacteria (additional file 3). The Clade A species/strains can also be distinguished from other cyanobacteria based upon a 15 aa conserved insert in the protein synthesis elongation factor-G that is specific for this clade [23].

### Signature proteins for the Clade B cyanobacteria

The Clade B comprises the majority of known cyanobacteria except the unicellular marine cyanobacteria (Clade C) and some deep branching cyanobacteria (see Fig. 1). This clade as defined in our work includes all of the species/strains from the orders *Chroococcales, Nostocales *and *Oscillatoriales *as well as the deeper branching cyanobacteria, *A. marina *and *Thermosyn. elongatus*. Of these latter cyanobacteria, *Acaryochloris *is unique in containing chlorophyll d as its primary photosynthetic pigment [45], whereas *Thermosynechococcus *is a unicellular thermophilic cyanobacterium [46]. Our analyses have identified 38 proteins that are uniquely shared by all or most of the species/strains from this clade. Two of the *Synechococcus *strains viz. PCC7002 and PCC7335, also consistently appeared in this group and of these *Synechococcus *PCC7002, for which sequence information was available from various cyanobacteria, branched with the *Chroococcales *in phylogenetic trees (Fig. 1 and additional file 2).

The branching position of *Syn. elongatus *(strains PCC 6301 and PCC 7942) is not resolved in phylogenetic trees [4,11,12,37,47]. It generally branches in between the Clades B and C species/strains in phylogenetic trees (Fig. 1, additional file 2) [23]. Our analyses have identified 22 proteins, which in addition to various Clade B cyanobacteria are also present in *Syn. elongatus *(Table 3b). It is known from earlier work that a number of cyanobacteria contain split DnaE protein due to the presence of intervening inteins [46,48,49]. Examination of DnaE gene/protein from various cyanobacteria indicates that the split DnaE proteins are found in all of the Clades B cyanobacteria as well as *Syn. elongatus*, whereas all other species/strains from clade A and C do not contain split DnaE [4](Gupta, R. S., results not shown). This rare genetic characteristic together with the various proteins in Table 3b suggests that *Syn. elongatus *and Clade B cyanobacteria probably shared a common ancestor exclusive of other cyanobacteria.

**Table 3:** Proteins Specific for Clade B Cyanobacteria

(a) Protein that are Uniquely found in All (or most) Clade B Cyanobacteria			
Protein	Function (length)	Protein	Function (length)
NP_439990/slr0723*^+^	Hypothetical (363)	NP_484675/all0631*^+^	Hypothetical (130)

NP_440199/slr0971	Hypothetical (451)	NP_484710/all0666*	Hypothetical (348)

NP_440305/slr0695^a^	Hypothetical (173)	NP_485162/all1119*^+^	Hypothetical (255)

NP_440382/sll1642*^+^	Hypothetical (163)	NP_485285/alr1242*	Hypothetical (221)

NP_440557/sll1573*	Hypothetical (104)	NP_485393/alr1350*^+^	Hypothetical (359)

NP_440936/slr0888*^+^	Hypothetical (168)	NP_485508/all1467*	Hypothetical (247)

NP_441490/sll1247*	Hypothetical (457)	NP_486386/alr2346*	Hypothetical (104)

NP_441696/slr1686*	Hypothetical (141)	NP_486393/asl2353*	Hypothetical (98)

NP_441913/sll1858*	Hypothetical (627)	NP_486647/asr2607*	Hypothetical (65)

NP_442061/slr0779*	Hypothetical (206)	NP_487221/all3181*	Hypothetical (322)

NP_442144/slr0217^+^	Hypothetical (140)	NP_487892/all3852	Hypothetical (281)

NP_484091/all0047*	Hypothetical (531)	NP_488032/asr3992*	photosystem II reaction center

NP_484127/alr0083*^+^	Hypothetical (137)	NP_488333/alr4293*	Hypothetical (163)

NP_484326/all0282*	Hypothetical(162)	NP_488559/all4519*	Hypothetical (104)

NP_484594/asl0550*	Hypothetical (72)	NP_488570/alr4530^2^	Hypothetical (388)

NP_484607/all0563	general secretion pathway protein (207)	NP_488633/all4593^a^	Hypothetical (434)

NP_484635/all0591*	Hypothetical (123)	NP_488729/all4689*	Hypothetical (169)

NP_484674/all0630*	Hypothetical (128)	NP_489127/alr5087*	Hypothetical (124)

**(b) Proteins Specific for clade B Cyanobacteria and also *Synechococcus elongates***			

NP_440371/ssl1918	Hypothetical (97)	NP_485176/alr1133*	Hypothetical (160)

NP_440821/slr1218	Hypothetical (158)	NP_485590/alr1550*	Hypothetical (119)

NP_441017/sll1757^+^	Hypothetical (292)	NP_486755/all2715*	Hypothetical (214)

NP_441155/sll1155	Hypothetical (67)	NP_486776/all2736*	Hypothetical (186)

NP_441519/slr1970*^+^	Hypothetical (173)	NP_487697/asr3657*	Hypothetical (120)

NP_441527/sll1884^+^	Hypothetical (374)	NP_488054/asl4014*	Hypothetical (98)

NP_441857/ssr0657	Hypothetical (103)	NP_488538/asr4498*	Hypothetical (86)

NP_442144/slr0217^+^	Hypothetical 140)	NP_488628/asr4588*	Hypothetical (68)

NP_442174/ssl0788^+^	Hypothetical (97)	NP_488797/all4757*	Hypothetical (116)

NP_442462/slr0845*	Hypothetical (190)	NP_488854/alr4814*	Hypothetical (162)

NP_484393/all0349*^+^	Hypothetical(138)	NP_489314/all5274*	Hypothetical (247)

* - Missing in 1-2 species

^+ ^Also present in *Synechococcus sp. PCC 7335*

^a ^A homolog showing significant similarity is also found in *Sorganum cellulosum*

Within Clade B, the cyanobacterial species/strains belonging to the orders *Nostocales, Oscillatoriales and Chroococcales *form a distinct clade (NOC clade) in phylogenetic trees (Fig. 1 and additional file 2). This clade has been referred to as the SPM clade in earlier work [4,47]. We have recently described a number of conserved indels in important proteins (viz. a 19 aa insert in DnaE protein, a 13 aa deletion in GDP-mannose pyrophosphorylase and a 22 aa insert in NAD(P)H-quinone oxidoreductase subunit D) that are distinctive characteristics of this clade of cyanobacteria [23]. In the present work, we have identified 9 proteins (Table 4a) that are also uniquely present in all of the species/strains from the NOC clade of cyanobacteria. In addition, 33 other proteins listed in the additional file 4 are also specific for the NOC clade, but they are missing in some species/strains. Within the NOC clade, species/strains belonging to the orders *Nostocales *and *Oscillatoriales *exhibit a closer relationship in phylogenetic trees (Fig. 1 and additional file 2). A 4 aa deletion in the translation initiation factor IF-2 is also uniquely shared by various sequenced cyanobacterial species/strains from these two orders [23]. In this study, we have come across 22 proteins that are specifically present in various sequenced species/strains from these two orders of cyanobacteria (Table 4b), providing further support that these two groups are more closely related.

**Table 4:** Proteins Specific for Different Groups within Clade B Cyanobacteria

(a) Proteins Specific for Nostocales, Oscillatoriales and Chroococcales (NOC) Orders			
**Protein**	**Function (length)**	**Protein**	**Function (length)**

NP_441847/sll0360^#^	Hypothetical (277)	NP_486936/asr2896	Hypothetical (63)

NP_484828/asr0785	Hypothetical (60)	NP_488368/asl4328	Hypothetical (68)

NP_485335/all1292	Hypothetical (142)	NP_488902/asl4862	Hypothetical (77)

NP_485350/asr1307	Hypothetical (78)	NP_488971/all4931	Hypothetical (225)

NP_485586/alr1546	Hypothetical (170)		

**(b) Proteins Specific for Nostocales and Oscillatoriales Orders**			

NP_484145/alr0101	Hypothetical (258)	NP_485811/all1771	Hypothetical (238)

NP_484259/all0215	Hypothetical (212)	NP_486433/alr2393	Hypothetical (343)

NP_484503/all0459*	Hypothetical (119)	NP_486508/asr2468*	Hypothetical (76)

NP_484625/asr0581*	Hypothetical (76)	NP_486828/all2788*	Hypothetical (146)

NP_484724/asr0680*	Hypothetical (94)	NP_487523/asr3483*	Hypothetical (64)

NP_484725/alr0681*	Hypothetical (115)	NP_488294/all4254^×^	Hypothetical (398)

NP_485091/asr1048*	Hypothetical (65)	NP_488340/all4300*	Hypothetical (227)

NP_485092/asr1049*	Hypothetical (88)	NP_488754/alr4714	Hypothetical (232)

NP_485286/asl1243*	Hypothetical (72)	NP_488903/alr4863	Hypothetical (999)

NP_485748/all1708*	Hypothetical (200)	NP_489130/all5090	Hypothetical (162)

NP_486432/alr2392*	filament integrity protein (179)	NP_489162/all5122	Hypothetical (119)

**(c) Proteins specific for Chroococcales**			

BAA10649/slr0111	hypothetical (173)	BAA17589/sll1268	hypothetical(517)

BAA10763	cytochrome b6-f complex subunit (36)	BAA17704/sll1755	hypothetical(407)

BAA16770/slr1107	hypothetical(444)	BAA18427/slr0960	hypothetical(146)

BAA17546/ssr2406	hypothetical(74)	BAA18451/sll1531	hypothetical(608)

**(d) Proteins specific for Nostocales**^+^			

NP_48404/all0002	Hypothetical (245)	NP_485976/asl1936	Hypothetical (81)

NP_484071/asl0027	Hypothetical (81)	NP_485977/asl1937	Hypothetical (83)

NP_484141/asl0097	Hypothetical (51)	NP_486406/alr2366	Hypothetical (118)

NP_484220/asl0176	Hypothetical (87)	NP_486414/alr2374	Hypothetical (129)

NP_484351/all0307	Hypothetical (114)	NP_486562/alr2522	Hypothetical (141)

NP_484421/alr0377	Hypothetical (153)	NP_486815/alr2775	Hypothetical (249)

NP_484504/asr0460	Hypothetical (81)	NP_487185/all3145	Hypothetical (122)

NP_484505/asr0461	Hypothetical (96)	NP_487215/alr3175	Hypothetical (264)

NP_484526/asr0482	Hypothetical (64)	NP_487290/asr3250	Hypothetical (69)

NP_484616/asl0572	Hypothetical (75)	NP_487319/asr3279	Hypothetical (64)

NP_484758/asl0715	Hypothetical (56)	NP_487408/asr3368	Hypothetical (75)

NP_484822/asl0779	Hypothetical (67)	NP_487429/asr3389	Hypothetical (75)

NP_484885/asl0842	Hypothetical (80)	NP_487760/alr3720	Hypothetical (129)

NP_484898/asr0855	Hypothetical (83)	NP_487950/alr3910	Hypothetical (252)

NP_484966/asr0923	Hypothetical (67)	NP_487957/alr3917	Hypothetical (447)

NP_485022/all0979	Hypothetical (220)	NP_488113/all4073	Hypothetical (121)

NP_485048/asr1005	Hypothetical (80)	NP_488149/all4109	Hypothetical (235)

NP_485180/alr1137	Hypothetical (107)	NP_488157/all4117	Hypothetical (411)

NP_485189/alr1146	Hypothetical (847)	NP_488392/asr4352	Hypothetical (65)

^# ^also found in one of the clade A cyanobacteria

* missing in 1-2 species/strains

^+^Additional proteins that are specific for Nostocales are listed in the Additional file 5.

Within Clade B, the heterocyst-forming cyanobacteria form a monophyletic group (subclass Nostocophycidae) [6,10,47,50]. We recently described two conserved indels (a 4 aa insert in the PetA protein, a precursor of the apocytochrome f, and a 5 aa insert in the ribosomal protein S3) that are specific for these bacteria [23]. In the present work, blast searches on the genome of *Nostoc sp. PCC7120 *have identified 65 proteins that are uniquely shared by all of the sequenced *Nostocales *species/strains (*Nostoc, Anabaena and Nodularia*) (Table 4d and additional file 5). Fifty-eight additional protein listed in the additional file 5 are also specific for this order, but they are missing in 1-2 species/strains. These proteins provide potential molecular signatures for the *Nostocales *order (Nostocophycidae subclass).

The cyanobacteria such as *Synechocystis, Microcystis*, *Crocosphaera *and *Cyanothece*, belonging to the order *Chroococcales*, form another well-defined clade in phylogenetic trees (see Fig. 1 and additional file 2) [4,11,12,37,47]. A 1 aa insert in a highly conserved region of the RecA protein is also specific for these cyanobacteria [23]. This insert is also present in *Synechococcus sp. PCC7002*, which branches with this clade in the phylogenetic trees (see Fig. 1 and additional file 2) [4,47]. In this work, we have identified 8 proteins that are uniquely present in various sequenced *Chroococcales *species/strains (Table 4c). The evolutionary stages where the genes for these proteins have likely evolved are indicated in the interpretive diagram (Fig. 2).

### Signature proteins for the Clade C Cyanobacteria

**Figure 3:**
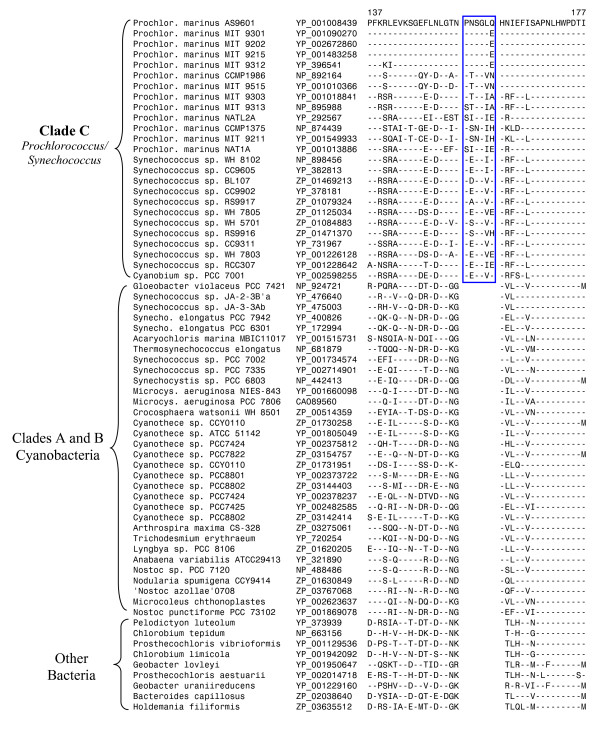
**Partial sequence alignment of flavoprotein showing a 6 aa conserved insert (boxed) that is specific for the Clade C cyanobacteria**. Dashes (-) in this and all other sequence alignments indicate identity with the amino acid on the top line. The numbers on the top indicate the position of the sequence in the species on the first line. The absence of this insert in all other cyanobacteria and other phyla of bacteria provide evidence that this indel is an insert in the Clade C.

The Clade C is comprised of different strains/isolates of marine *Prochlorococcus *and *Synechococcus *[40,41,51-53]. We have recently described a number of conserved indels in widely distributed proteins that are specific for all of the species/strains from Clade C [23]. These signatures include a 3 aa insert in the RNA polymerase beta subunit, a 2 aa insert the proteins KsgA, a 6 aa insert in tyrosyl-tRNA synthetase, a 2 aa insert in the tRNA (guanine-N1-)-methyltransferase, a 1 aa insert in the RNA polymerase β' subunit and a 12 aa insert in the DNA polymerase I [23]. These signature indels are not found in the Clades A or B cyanobacteria or other phyla of bacteria. Additionally, they are also absent in *Syn. elongatus *as well as *Synechococcus sps*. PCC7002 and PCC7335. Another example of a signature insert that is specific for Clade C species/strains is presented in Fig. 3. In this case, a 6 aa insert in a flavoprotein is commonly present in all Clade C species/strains, but absent from all other cyanobacteria as well as other bacteria. This latter observation indicates that this indels is an insert in the Clade C species/strains. Interestingly, this insert and also several of the other Clade C signature indels are also present in *Cyanobium *sp. PCC7001 (Fig. 3), supporting its placement within the Clade C (Fig. 2) [4,15].

Our blast analyses on proteins from the genomes of *Synechococcus sp. WH8102, Prochlorococcus sp. MIT9215 *and *Pro. marinus subsp. marinus str. CCMP1375 *have identified 60 proteins that are uniquely shared by virtually all of the species/strains from Clade C cyanobacteria (Table 5a). These signature proteins provide further evidence and molecular markers indicating the distinctness of Clade C. Eight additional proteins in Table 5b are also specific for Clade C cyanobacteria, but they are absent in all of the low B/A ecotype *Prochlorococcus *strains, indicating that the genes for these proteins were lost from a common ancestor of the low B/A clade.

**Table 5:** Proteins Specific for the Clade C Cyanobacteria (*Synechococcus/Prochlorococcus*)

Protein	Function (length)	Protein	Function (length)
NP_874427/Pro0033	predicted membrane protein (87)	YP_001483584	Hypothetical (114)

NP_874433/Pro0039	predicted membrane protein (203)	YP_001483784	Hypothetical (60)

NP_874460/Pro0066	predicted membrane protein (128)	YP_001483792^+^	Hypothetical (116)

NP_874461/Pro0067	Hypothetical (154)	YP_001483839	Hypothetical(75)

NP_874496/Pro0102	Hypothetical (121)	YP_001484024	Hypothetical (67)

NP_874497/Pro0103	Hypothetical (76)	YP_001484070	Hypothetical (96)

NP_874503/Pro0109	Hypothetical (127)	YP_001484558	Hypothetical(70)

NP_874769/Pro0375	Hypothetical (128)	YP_001484735	Hypothetical(136)

NP_874827/Pro0433	Hypothetical (148)	YP_001484929	Hypothetical (89)

NP_874971/Pro0578	Hypothetical (104)	YP_001484936	Hypothetical (237)

NP_875238/Pro0846	Hypothetical (135)	YP_001485057	Hypothetical(88)

NP_875250/Pro0858	Hypothetical (116)	YP_001485093	Hypothetical (172)

NP_875290/Pro0898	Hypothetical (75)	YP_001485151^+^	Hypothetical (139)

NP_875352/Pro0960	Hypothetical (76)	NP_875191/Pro0799*	Hypothetical (234)

NP_875454/Pro1062	Hypothetical (189)	NP_875240/Pro0848*	membrane protein/(99)

NP_875462/Pro1070	dihydroneopterin aldolase (127)	NP_875270/Pro0878*	Hypothetical (62)

NP_875555/Pro1163	predicted protein family PM-1 (67)	YP_001483575*	Hypothetical(71)

NP_875594/Pro1202	Hypothetical (81)	YP_001483809*^+^	Hypothetical(116)

NP_875635/Pro1243	Hypothetical (193)	YP_001483828*	Hypothetical(122)

NP_876135/Pro1744	Hypothetical (206)	YP_001483924*	Hypothetical(502)

NP_876152/Pro1761	Hypothetical (98)	NP_875468/Pro1076*	Hypothetical (88)

NP_876219/Pro1828	Hypothetical (100)	NP_875511/Pro1119*	Predicted protein with signal (144)

YP_001010165	Hypothetical(121)	NP_875732/Pro1341*	Hypothetical (88)

YP_001483235	type II secretion system (149)	NP_876151/Pro1760*	Hypothetical (152)

YP_001483304	Hypothetical (100)	NP_876229/Pro1838*	Hypothetical (171)

YP_001483312	Hypothetical (87)	YP_001483988*	Hypothetical(70)

YP_001483445	Hypothetical(72)	YP_001484266*	Hypothetical(195)

YP_001483588	TIR domain-containing protein (82)	YP_001483537*	possible Pollen allergen (139)

YP_001483568	hypothetical (102)	YP_001483448	Hypothetical (42)

YP_001484489	hypothetical (85)	YP_001484000	hypothetical (80)

**(b) Proteins Specific for Clade C which are missing in Low B/A ecotype Prochlorococcus strains**^#^			

NP_875075/Pro0683*	Predicted protein family PM-3 (178)	NP_875154/Pro0762	Hypothetical (127)

NP_874434/Pro0040*	Hypothetical (119)	NP_875509/Pro1117	Hypothetical (181)

NP_874631/Pro0237	Hypothetical (102)	NP_875611/Pro1219*	Predicted protein family PM-3 (195)

NP_875013/Pro0621*	predicted protein family PM-3 (167)	NP_876129/Pro1738*	Predicted dehydrogenase (273)

* - Missing in 1-2 species

^+^Also present in *Synechococcous elongatus*

Several of these proteins are also present in *Cyanobium sp. PCC7001 *and *Paulinella chromatophora*

^#^Low B/A ecotype clade is comprised of the following Prochlorococcus strains: Pro. marinus AS9601, Pro. marinus MIT9215, Pro. marinus MIT9301, Pro. marinus MIT9312, Pro. marinus MIT9515, Pro. marinus CCMP1986

As noted earlier, in phylogenetic trees, the branching position of *Syn. elongatus *is not resolved. In our analyses, we have come across only 3 proteins (marked with ^+ ^in Table 5a) that are uniquely found in Clade C species/strains as well as *Syn. elongatus*. This is in contrast to 22 proteins that are uniquely shared by Clade B cyanobacteria and *Syn. elongatus *(Table 3b). These observations in conjunction with the unique presence of split DnaE genes in Clade B cyanobacteria and *Syn. elongatus *make a strong case that *Syn. elongatus *is more closely related to the Clade B cyanobacteria than to the Clade C species/strains.

**Figure 4:**
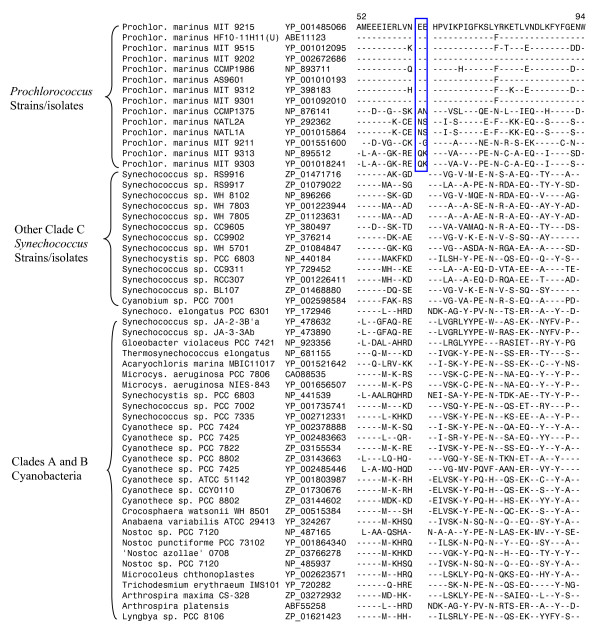
**Partial sequence alignment of heme oxygenase showing a 2 aa insert (boxed) that is uniquely present in all sequenced *Prochlorococcus *strains, but not found in any other cyanobacteria**. This insert provides evidence that *Prochlorococcus *strains are monophyletic and shared a common ancestor.

The two genera, *Prochlorococcus *and *Synechococcus*, which make up most of the Clade C cyanobacteria, differ from each other in important respects, particularly with regard to the main pigments in their light harvesting systems [40,41]. In contrast to various *Synechococcus *strains/isolates and most other cyanobacteria, which contain chlorophyll **a **and phycobiliproteins as the major pigments in their photosynthetic systems, all *Prochlorococcus *strains/isolates utilize divinyl chlorophyll **a **and both mono and divinyl chlrophyll **b **as the main pigments in their light-harvesting systems [40,41]. Further, while *Synechococcus *isolates are ubiquitous in different aquatic environments including estuarine, coastal and offshore waters [53], *Prochlorococcus *strains are mainly found in warm oligotrophic oceanic settings [40]. Among the sequenced cyanobacteria, *Prochlorococcus *strains/isolates have the smallest genomes (see Table 1). Although *Prochlorococcus *are indicated to be polyphyletic in phylogenetic analyses (with strains MIT 9303 and MIT 9313 branching within the *Synechococcus *strains/isolates; see Fig. 1 and additional file 2) [12,23,33], our blast searches have identified 19 proteins that are uniquely shared by all or most of the *Prochlorococcus *strains (Table 6b). These results indicate that despite their polyphyletic branching in phylogenetic trees, all *Prochlorococcus *strains/isolates form a monophyletic clade, which is in accordance with their distinctive photosynthetic pigments composition. In this work, we also describe a 2 aa conserved insert in the protein heme oxygenase that is also exclusively present in various *Prochlorococcus *strains (Fig. 4). The unique presence of this insert in various *Prochlorococcus *strains provides further evidence that this group is monophyletic. The enzyme heme oxygenase, which contains this conserved insert, plays an important role in the biosynthesis of photosynthetic pigments phyto-chromobilin and phycobilins [54]. Because *Prochlorococcus *are unique in terms of their photosynthetic pigment composition, it is of much interest to determine the functional significance of this conserved indel.

**Table 6:** Proteins specific for the Main Groups of Clade C Cyanobacteria

(a) Proteins Specific for the Clade C cyanobacteria^+ ^except *Prochlorococcus*			
**Protein**	**Function (length)**	**Protein**	**Function (length)**

NP_896793/SYNW0700	Hypothetical (76)	NP_897761/SYNW1668	Hypothetical (181)

NP_896942/SYNW0849*	Hypothetical (120)	NP_898450/SYNW2361*	Hypothetical (129)

NP_897039/SYNW0946	Hypothetical (139)	NP_896879/SYNW0786*	Hypothetical (107)

NP_896623/SYNW0528*	Hypothetical(94)	NP_896904/SYNW0811*	Hypothetical (81)

NP_896827/SYNW0734	Hypothetical(152)	NP_897398/SYNW1305*	Hypothetical(78)

NP_897338/SYNW1245*	Hypothetical(95)	NP_897599/SYNW1506	Hypothetical(221)

NP_897228/SYNW1135*	Hypothetical(139)	NP_897875/SYNW1784	Hypothetical(150)

**(b) Proteins Specific for *Prochlorococcus***			

YP_001483307	hypothetical (58)	YP_001484319*	hypothetical (94)

YP_001483938*	hypothetical (109)	YP_001484350	hypothetical (104)

YP_001483942	hypothetical (75)	YP_001484353*	hypothetical (68)

YP_001483946	hypothetical (88)	YP_001484529*	hypothetical (99)

YP_001483975*	hypothetical (99)	YP_001484536*	hypothetical (42)

YP_001483996*	hypothetical (51)	NP_875788*	hypothetical (81)

YP_001484105*	hypothetical (64)	YP_001483983*	hypothetical (96)

YP_001484131	hypothetical (61)	YP_001484474*	hypothetical (79)

YP_001484828	hypothetical (55)	YP_001484870	hypothetical (142)

YP_001483822	hypothetical (44)		

* Missing in 1-2 strains/isolates

^+ ^These proteins are primarily present in various Synechococcus species/strains that are part of Clade C (see Figs. 1 and 2). However, *Synechococcus *genus is not monophyletic and many *Synechococcus *strains group with Clade and B (viz. *Synechococcus sp. PCC7002*, Synechococcus sp. PCC7335, Synechococcus sp. JA-3-3Ab and JA-2-3B'a) and these proteins are absent in those strains. Besides *Synechococcus*, homologs of many of these proteins are also found in *Cyanobium sp. PCC7001 *as well as in *Paulinella chromatophora*, indicating that these species may also belong to the Clade C cyanobacteria.

If *Prochlorococcus *strains/isolates form a monophyletic lineage, then one expect that other cyanobacteria that are part of Clade C might also share many unique proteins in common. Indeed, our blast searches have identified 14 proteins that are uniquely present in various other cyanobacteria (mostly *Synechococcus *strains) that are part of Clade C (Table 6a). It should be mentioned that for several of these proteins, blast hits indicating significant similarity are also found for *Cyanobium sp. PCC7001 *and *Paulinellla chromatophora*, indicating that these cyanobacteria are also part of the Clade C. The grouping of *Cyanobium sp. PCC7001 *with Clade C is also supported by the conserved indel in the flavoprotein (see Fig. 3).

**Figure 5:**
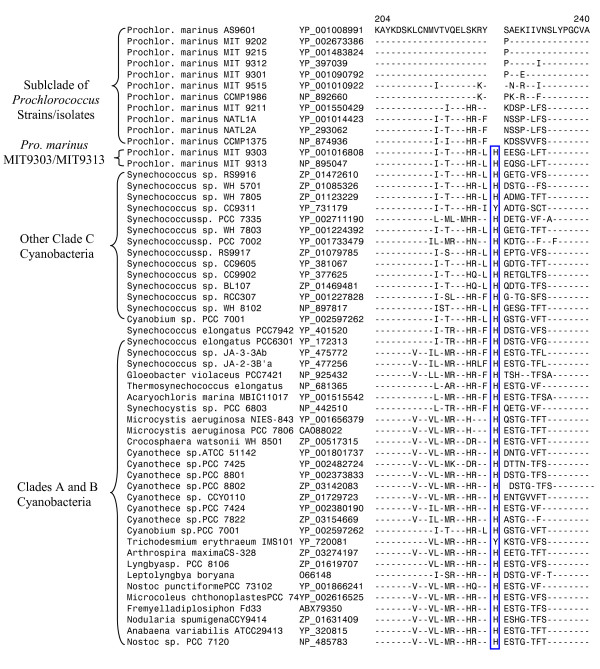
**Partial sequence alignment of the protein protochlorophyllide oxidoreductase showing a 1 aa deletion that is commonly shared by all *Prochlorococcus *strains except MIT 9303 and MIT 9313**. This indel provides evidence for the deep branching of these *Prochlorococcus *strains relative to all other strains.

As noted above, in phylogenetic trees based on concatenated protein sequences *Prochlorococcus *str. MIT9303 and MIT9313 branch within the various *Synechococcus *strains/isolates (Fig. 1 and additional file 2). Earlier phylogenetic studies by Rocap et al. [41] based on the 16S-23S rDNA spacer region indicate that these two strains (high B/A clade IV) form the deepest branching isolates of this genus. Further, in contrast to other sequenced *Prochlorococcus *strains, whose G+C content range from 30-39%, the strains MIT9303 and MIT9313 have much higher G+C content (~50%) (see Table 1). Our blast analyses, in addition to identifying many proteins that are unique to various *Synechococcus *strains/isolates, have also identified 22 proteins that are specifically present in all of the Clade C *Synechococcus *strains as well as in *Prochlorococcus *MIT9303 and MIT9313 (additional file 6a). At the same time, we have come across 37 proteins that are uniquely found in all other sequenced *Prochlorococcus *strains, but which are missing in MIT9303 and MIT9313 (additional file 6b). In addition, we have also identified a 1 aa deletion in a conserved region of the protein protochlorophyllide oxidoreductase (POR) that is uniquely shared by all other *Prochlorococcus *strains except MIT9303 and MIT9313 (Fig. 5). The enzyme POR is responsible for catalyzing light driven reduction of protochlorophyllide to chlorophyllide - a key regulatory reaction in the chlorophyll biosynthetic pathway [55]. Hence, it is again of much interest to understand the functional significance of this conserved indel. The rare genetic change leading to this indel likely occurred in a common ancestor of various *Prochlorococcus *strains after the branching of MIT9303 and MIT9313 (Fig. 2). These observations, in conjunction with the branching pattern of these strains in phylogenetic trees, provide evidence that these two *Prochlorococcus *strains comprise the deepest branching group (high B/A clade IV) [41] within the *Prochlorococcus *genus, exhibiting closest relationship to the *Synechococcus *strains/isolates.

Earlier studies have led to the division of *Prochlorococcus *strains/isolates into two physiologically distinct groups (high B/A and low B/A ecotypes), based upon the ratios of chlorophyll **b **and **a2 **in their light-harvesting systems and their ability to grow at different light intensities [40,41,56]. Of these two groups, strains from the high B/A ecotype, which have larger ratio of chlorophyll **b/a**_2 _are able to grow at extremely low irradiance, whereas those from the low- B/A ecotype containing lower ratio of chlorophyll **b/a**_2 _are unable to grow under these conditions. The low- B/A ecotype strains instead are adapted to growth at high light intensities, where the growth of high B/A ecotype strains is inhibited. The strains from these two ecotypes also differ in terms of their sensitivity to copper and their ability to use nitrite or nitrate as nitrogen sources [41,57]. In phylogenetic trees, the low B/A ecotype *Prochlorococcus *isolates (viz. MIT9515, CCMP1986, MIT9312, MIT9215, MIT9301 and AS9601) formed a distinct subclade that was well separated from all other Clade C species/strains by a long-branch and 100% bootstrap score (Fig. 1 and additional file 2)[23,41]. We have also described two conserved indels (viz. a 5 aa deletion in leucyl-tRNA synthetase and 1 aa insert in the Ffh protein) that are uniquely shared by all of the low B/A ecotype *Prochlorococcus *strains [23]. In the present work, we have identified 67 proteins that are exclusively found in all of the sequenced strains from the low B/A ecotype clade (additional file 7a). Seventy-two proteins listed in the additional file 7b are also specific for this clade, but they are missing in 1-2 of the strains/isolates. These signature proteins and indels together with the distinct branching of the low B/A strains in phylogenetic trees provide strong evidence that this group of *Prochlorococcus *strains are phylogenetically, physiologically and molecularly distinct from all other *Prochlorococcus *strains. Based upon species distribution patterns of various cyanobacteria-specific proteins, evolutionary stages where the genes for these proteins likely evolved are indicated in the interpretive diagram in Fig. 2.

## Discussion and Conclusions

In this work, we have used a combination of phylogenomic and signature proteins based approaches to elucidate the evolutionary relationships among cyanobacteria. Phylogenetic trees were initially constructed for 44 cyanobacteria based on concatenated sequences for 44 widely distributed proteins present in various cyanobacteria. The branching pattern of cyanobacteria in these trees was very similar to that observed in other recent studies based on different large sets of proteins for smaller numbers of cyanobacteria [4,11,12]. In all of these trees a number of distinct clades of cyanobacteria are consistently observed. However, the main focus of the present work was on comparative analyses of cyanobacterial genomes to identify unique sets of genes/proteins that are limited to particular groups of cyanobacteria, corresponding to various phylogenetically identified clades. This work complement our recent studies, where a comparative genomic approach was employed to identify >40 conserved indels in widely distributed proteins that are also specific for the same groups/clades of cyanobacteria [23].

Recent analyses of genomic sequences have revealed that whole proteins that are limited to different monophyletic clades are present at different phylogenetic depths [26-28,43,44,58,59]. Unlike ORFan proteins, which are unique to a given species or a strain and are subject to rapid gene loss [44,60,61], these lineage-specific proteins are retained in a conserved state by all or most species/strains from a given clade, indicating that they are conferring selective advantage to species from these clades [28,58,62]. Although the mechanism responsible for the evolution or acquisition of genes for these proteins is unclear [28,61], their specific presence in different clades indicates that the genes for these proteins first evolved (or introduced) in a common ancestor of these clades followed by their retention by various descendents of these clades. Because of their clade specificity, these lineage specific-proteins or conserved signature proteins (CSPs) provide valuable molecular markers for these clades [26-28,43,59]. Our recent analyses of CSPs from several major groups of bacteria (viz. alpha proteobacteria, epsilon proteobacteria, gamma proteobacteria, chlamydiae, *Bacteroidetes-Chlorobi *and *Actinobacteria*) provide evidence that the species distribution of most of these CSPs show high degree of concordance with different clades in the phylogenetic trees [25-27,42,63,64]. This inference is strongly reinforced by the results of present study, where most of the identified CSPs correspond to well-defined clades in the phylogenetic trees.

It should be mentioned that in our analyses we have not come across significant numbers of CSPs that support alternate groupings i.e. where the proteins are commonly shared by various species/strains from clades that are phylogenetically unrelated (e.g. *Nostocales *and Clade C, or *Oscillatoriales *and Clade C). However, one commonly observed pattern is that if two clades are close to each other in phylogenetic trees, but their branching is not clearly resolved (i.e. weakly supported by bootstrap scores), then in addition to observing many proteins that are unique to each of these two clades, several proteins that are commonly shared by them are also observed. This could be due to either that genes for many of these proteins probably evolved in a common ancestor of these clades prior to their becoming phylogenetically distinct or due to lateral gene transfers among closely related taxa [13,65]. Nevertheless, our results that most of these proteins are distinctive characteristics of phylogenetically well-defined monophyletic clades strongly suggest that their species distribution has not been significantly affected by lateral gene transfers, which is indicated to be very common in cyanobacteria [13,66].

When a protein is confined to only a certain group of species/strains, then based upon this information alone, it is difficult to determine whether the group of species containing this protein form a clade in the phylogenetic sense or not. To properly evaluate the results of such studies, it is necessary to carry out these studies in conjunction with phylogenetic as well as other forms of analyses (e.g. studies based on conserved indels), where it is possible to establish a rooted relationship among different groups or taxa under consideration [23,26,59]. Based on these studies, if a given protein is uniquely found in all or most of the species from a well-defined monophyletic clade, and generally no where else, then the simplest and most parsimonious explanation for this is that the gene for this protein first appeared in a common ancestor of this group and then passed on vertically to its various descendants [17,20,67]. We have interpreted the results of species distribution of various unique proteins based on this minimal assumption. Based on this interpretation, various identified signature proteins or CSPs could be regarded as molecular synapomorphies that are specific for different clades of cyanobacteria.

The branching order and interrelationships among cyanobacteria that emerges based upon all of these different approaches is shown in Fig. 2. All of these approaches indicate that a clade consisting of *Gloebacter *and the *Synechococcus *strains JA-3-3Ab and JA2-3-B'a (Clade A) forms the deepest branching lineage within cyanobacteria. A large number of sequenced cyanobacteria correspond to marine unicellular *Synechococcus *and *Prochlorococcus *strains (Clade C). We have identified numerous proteins and conserved indels that are specific for this clade. Although *Synechococcus *and *Prochlorococcus *strains do not form monophyletic clusters in phylogenetic trees, the shared presence of many novel proteins as well as some conserved indels by various *Prochlorococcus *strains provide evidence that this group is monophyletic. The unique pigments that are found in the light harvesting system of *Prochlorococcus *also support their distinctness from other cyanobacteria. The monophyletic grouping of marine unicellular *Synechococcus *strains/isolates based upon these molecular and biochemical characteristics is at variance with their polyphyletic branching in different phylogenetic trees (see Fig. 1, additional file 2) [4,11,23]. This discordance could be explained by either lateral migration of genes responsible for these characteristics [11,13,33,68], or due to inability of the phylogenetic trees to resolve the branching order among closely related species/strains. Among the *Prochlorococcus *strains, our analyses confirm that the strains corresponding to low B/A ecotype are distinct not only in physiological and phylogenetic terms [40,41,56], but that they also share large numbers of proteins that are unique to them. Several conserved indels that are specific for the low B/A ecotype clade have also been identified [23]. Recent study by Zhaxybayeva et al. [33] also provides evidence that the high-light adapted low B/A ecotype *Prochlorococcus *strains form a monophyletic clade, in contrast to the paraphyletic grouping of the low-light adapted (i.e. high B/A ecotype) *Prochlorococcus *spp. [33]. All of these observations make a strong case for the recognition of low B/A ecotype *Prochlorococcus *strains as a distinct taxonomic entity.

Within Clade B, many CSPs were identified that are specific for the *Nostocales *and *Chroococcales *orders. In addition, several other CSPs are uniquely present in the *Nostocales *and *Oscillatoriales *orders, or by the *Nostocales*, *Oscillatoriales *and *Chroococcales*. In recent work, a number of conserved indels that are unique to these orders of cyanobacteria have also been identified [23]. Although, the clade comprising of these cyanobacterial orders is not clearly resolved in phylogenetic trees [4,11], the shared presence of large numbers of novel CSPs as well as some conserved indels by these cyanobacteria strongly suggests that species/strains from these groups shared a common ancestor exclusive of other cyanobacteria and that this clade represents a deeper branching grouping within cyanobacteria. The results presented here also suggest that *Syn. elongatus *is more closely related to Clade B in comparison to either clade A or C of cyanobacteria.

The signature proteins and conserved indels for different cyanobacterial clades that are described in this work and in our recent studies [23] provide novel and powerful means for understanding cyanobacterial phylogeny and taxonomy. Based on these molecular markers, all of the main clades of cyanobacteria can now be identified and circumscribed in molecular terms. These signature proteins and indels should also prove useful for the identification and assignment of cyanobacterial species/strains to specific clades based upon the presence or absence of various signature indels or CSPs. Because many of these CSPs, or proteins containing the conserved indels, are highly conserved, degenerate PCR primers could be readily designed to sequence the corresponding genes/proteins from any given cyanobacteria. The assignment of any species/strains into a given clade by this approach is based upon several independent signatures that provide complementary information. Some of these signatures serve to exclude a given species/strains from particular groups or clades, whereas others point to its inclusion in more and more specific clades. Blast searches with these cyanobacteria-specific CSPs should also prove useful in determining the presence or absence of different groups of cyanobacteria in metagenomic sequences [69]

Most of the cyanobacterial signature proteins identified in this work are of unknown functions. However, the retention of these genes by all cyanobacteria from the indicated clades strongly suggests that these proteins perform important functions in these groups of cyanobacteria [70-72]. Likewise, our recent work shows that the conserved indels in protein sequences are also essential for the group or clade of species where they are found [73]. Hence, further work on understanding the cellular functions of these cyanobacterial signature proteins and signature indels should be of great interest. These studies should provide valuable insights regarding biochemical and physiological characteristics that are unique to different clades of cyanobacteria [64,74-76].

## Methods

### Phylogenetic/phylogenomic analyses

Phylogenetic analyses were carried out on a set of 44 proteins involved in important housekeeping functions that are present in most organisms (see Additional file 1) [35]. Blast searches with these proteins revealed that their homologs were present in all 34 sequenced cyanobacterial genomes (listed in Table 1), the two outgroup species (*Bacillus subtilis *and *Staphylococcus aureus*), as well as 10 other cyanobacteria (viz. *Crocosphaera watsonii WH8501, Cyanothece sp. CCY0110, Lyngbya sp. PCC8106, Microcystis aeruginosa PCC7806, Nodularia spumigena CCY9414, Syenchococcus sp. WH5701, Syenchococcus sp. BL107, Syenchococcus sp. RS9917, Syenchococcus sp. RS9916 *and *Syenchococcus sp. WH7805*). Hence, sequence information for all of these cyanobacteria was included in our analyses. The multiple sequence alignments for these proteins were created using the ClustalX 1.83 program [77] and they were concatenated into a single large file. This unedited sequence alignment was imported into the Gblocks 0.91b program to remove poorly aligned regions [78]. This program was used with default settings except that allowed gap position parameter was changed to half. The resulting final alignment of 16834 amino acid sites was used for phylogenetic analyses. A neighbour-joining (NJ) tree based on 1000 bootstrap replicates was constructed by the Kimura model [79] using the TREECON 1.3b program [80]. The maximum-likelihood (ML) analysis was carried out using the WAG+F model with gamma distribution of evolutionary rates with four categories using the TREE-PUZZLE program with 10000 puzzling steps [81].

### Identification of proteins and conserved indels that are specific for Cyanobacteria

The Blastp searches were carried out on each ORF in the genomes of *Synechococcus sp. WH8102, Synechocystis sp. PCC6803, Nostoc sp. PCC7120, Synechococcus sp. JA-3-3Ab, Prochlorococcus sp. MIT9215 *and *Prochlorococcus marinus subsp. marinus str. CCMP1375 *to identify proteins that are uniquely present in various clades of cyanobacteria seen in the phylogenetic trees (Fig. 1). The blast searches were performed against all organisms (i.e. non-redundant (nr) database) using the default parameters, without the low complexity filter [82]. The proteins that were of interest were those where either all significant hits were from the indicated groups of cyanobacteria, or which involved a large increase in E values from the last hit belonging to a particular clade to the first hit from any other bacteria/cyanobacteria and the E values for the latter hits were >1e^-04^, indicating weak similarity that could occur by chance. Higher E values are often significant for smaller proteins as the magnitude of the E value depends upon the length of the query sequence [82]. Hence, the lengths of the query proteins and those of various hits were also taken into consideration when analyzing the results of these studies. In most cases, the lengths of various significant hits were very similar to those of the query proteins. Some proteins, which in addition to cyanobacteria were also found in the plants/plastids, or in an isolated species from some other groups (noted appropriately), were also retained. The proteins, which were uniquely found in a given species or strain were not examined in this work. For all cyanobacterial proteins that are specific for various clades or subgroups, their accession numbers, any information regarding cellular functions, and protein lengths, were tabulated and are presented. Identification of new conserved indels that are specific for cyanobacterial clades was carried out as described in our earlier work [22,23].

## Authors' contributions

The initial Blastp searches on various cyanobacterial genomes were carried out by RSG with the computer assistance provided by Venus Wong. DWM analyzed the results of these searches to identify various group-specific proteins. All of these results were checked by RSG. DWM also generated a concatenated alignment of various cyanobacteria. RSG was responsible for carrying out the phylogenetic studies and for identification of conserved indels that are reported here. RSG also directed this study and wrote the manuscript, which was read and approved by all authors.

## Supplementary Material

Additional file 1 **List of proteins used in phylogenetic analyses**. The information for various proteins regarding their lengths, accession numbers, Gene bank IDs, locus tag for Nostoc sp. PCC7120 and COG groups is provided. Click here for file

Additional file 2 **Neighbour-joining tree for the sequenced Cyanobacteria**. A neighbour-joining, bootstrapped tree for 44 cyanobacteria based on concatenated sequences for 44 proteins listed in additional file 1. The sequences for *B. subtilis *and *S. aureus *were used to root this tree. Click here for file

Additional file 3 **Proteins that are specific for the Clade A of Cyanobacteria.**. All of the proteins listed in this Table are specific for Clade A, which consists of *G. violaceus *and *Synechococcus sps. JA-3-3Ab *and *JA-2-3B'a*. Click here for file

Additional file 4 **Proteins specific for the *Nostocales, Oscillatoriales *and *Chroococcales *orders.**. As above Click here for file

Additional file 5 **Proteins specific for the *Nostocales *order.**. As above Click here for file

Additional file 6 **Clade C proteins showing anomalous behavior of *Pro. marinus MIT9303 *and *MIT9313*.**. This table describes two sets of proteins: (a) Proteins that are specific for Clade C *Synechococcus *strains/isolates that are also found in *Pro. marinus MIT9303 *and *MIT9313 *and (b) Proteins specific for various other Prochlorococcus marinus strains/isolates, but which are missing in *Pro. marinus MIT9303 *and *MIT9313*. Click here for file

Additional file 7 Proteins specific for the Low B/A ecotype *Pro. marinus *strains/isolates. Click here for file

## References

[B1] Rippka R Deruelles J Waterbury JB Herdman M Stanier RY Generic assignments, strain histories and properties of pure cultures of cyanobacteria J Gen Microbiol 1979 111 1 61

[B2] Kondratieva EN Pfennig N Truper HG Balows A, Truper HG, Dworkin M, Harder W, Schleifer KH The Phototrophic Prokaryotes The Prokaryotes 1992 New York: Springer-Verlag 312 330

[B3] Castenholz RW Boone DR, Castenholz RW Phylum BX. Cyanobacteria: Oxygenic Photosynthetic Bacteria Bergey's Manual of Systematic Bacteriology 2001 New York: Springer 474 487

[B4] Sanchez-Baracaldo P Hayes PK Blank CE Morphological and habitat evolution in the Cyanobacteria using a compartmentalization approach Geobiology 2005 3 145 165 10.1111/j.1472-4669.2005.00050.x

[B5] Wilmotte A Golubic S Morphological and genetic criteria in the taxonomy of Cyanophyta/Cyanobacteria Arhciv fur Hydrobiologie 1991 64 1 24

[B6] Wilmotte A Herdman M Boone DR, Castenholz RW Phylogenetic Relationships among the Cyanobacteria Based on 16S rRNA Sequences Bergey's Manual of Systematic Bacteriology 2001 New York: Springer 487 493

[B7] Maidak BL Cole JR Lilburn TG Parker CT Jr Saxman PR Farris RJ Garrity GM Olsen GJ Schmidt TM Tiedje JM The RDP-II (Ribosomal Database Project) Nucleic Acids Res 2001 29 173 174 10.1093/nar/29.1.173 11125082 PMC29785

[B8] Garrity GM Bell JA Lilburn TG Brenner DJ, Krieg NR, Staley JT The Revised Road Map to the Manual Bergey's Manual of Systematic Bacteriology, Part A, Introductory Essays 2005 2 New York: Springer 159 220 full_text

[B9] Oren A A proposal for further integration of the cyanobacteria under the Bacteriological Code Int J Syst Evol Microbiol 2004 54 1895 1902 10.1099/ijs.0.03008-0 15388760

[B10] Hoffmann L Nomenclature of Cyanophyta/Cyanobacteria: roundtable on the unification of the nomenclature under the Botanical and Bacteriological Codes Algological Studies 2005 117 13 29 10.1127/1864-1318/2005/0117-0013

[B11] Swingley WD Blankenship RE Raymond J Integrating Markov clustering and molecular phylogenetics to reconstruct the cyanobacterial species tree from conserved protein families Mol Biol Evol 2008 25 643 654 10.1093/molbev/msn034 18296704

[B12] Shi T Falkowski PG Genome evolution in cyanobacteria: the stable core and the variable shell Proc Natl Acad Sci USA 2008 105 2510 2515 10.1073/pnas.0711165105 18268351 PMC2268167

[B13] Zhaxybayeva O Gogarten JP Charlebois RL Doolittle WF Papke RT Phylogenetic analyses of cyanobacterial genomes: quantification of horizontal gene transfer events Genome Res 2006 16 1099 1108 10.1101/gr.5322306 16899658 PMC1557764

[B14] Oren A Stackebrandt E Prokaryote taxonomy online: challenges ahead Nature 2002 419 15 10.1038/419015c 12214210

[B15] Hoffmann L Komarek J kastovsky J System of Cyanoprokaryotes (Cyanobacteria) - State in 2004 Algological Studies 2005 95 1155 10.1127/1864-1318/2005/0117-0095

[B16] Gupta RS Griffiths E Critical Issues in Bacterial Phylogenies Theor Popul Biol 2002 61 423 434 10.1006/tpbi.2002.1589 12167362

[B17] Gupta RS Protein Phylogenies and Signature Sequences: A Reappraisal of Evolutionary Relationships Among Archaebacteria, Eubacteria, and Eukaryotes Microbiol Mol Biol Rev 1998 62 1435 1491 9841678 10.1128/mmbr.62.4.1435-1491.1998 PMC98952

[B18] Gupta RS The phylogeny of Proteobacteria: relationships to other eubacterial phyla and eukaryotes FEMS Microbiol Rev 2000 24 367 402 10.1111/j.1574-6976.2000.tb00547.x 10978543

[B19] Delwiche CF Kuhsel M Palmer JD Phylogenetic analysis of tufA sequences indicates a cyanobacterial origin of all plastids Mol Phylogenet Evol 1995 4 110 128 10.1006/mpev.1995.1012 7663757

[B20] Rivera MC Lake JA Evidence that eukaryotes and eocyte prokaryotes are immediate relatives Science 1992 257 74 76 10.1126/science.1621096 1621096

[B21] Griffiths E Gupta RS Phylogeny and shared conserved inserts in proteins provide evidence that Verrucomicrobia are the closest known free-living relatives of chlamydiae Microbiology 2007 153 2648 2654 10.1099/mic.0.2007/009118-0 17660429

[B22] Gupta RS Pereira M Chandrasekera C Johari V Molecular signatures in protein sequences that are characteristic of Cyanobacteria and plastid homologues Int J Syst Evol Microbiol 2003 53 1833 1842 10.1099/ijs.0.02720-0 14657112

[B23] Gupta RS Protein signatures (molecular synapomorphies) that are distinctive characteristics of the major cyanobacterial clades Int J Syst Evol Microbiol 2009 59 2510 2526 10.1099/ijs.0.005678-0 19622649

[B24] Palmer JD Delwiche CF Sotis DE, Soltis PE, Doyle JJ The origin and evolution of plastids and their genomes Molecular Systematics of Plants II DNA Sequencing 1998 Norwell, MA, USA. Kluwer Academic Publishers 375 409

[B25] Gao B Parmanathan R Gupta RS Signature proteins that are distinctive characteristics of Actinobacteria and their subgroups Antonie van Leeuwenhoek 2006 90 69 91 10.1007/s10482-006-9061-2 16670965

[B26] Gupta RS Lorenzini E Phylogeny and molecular signatures (conserved proteins and indels) that are specific for the Bacteroidetes and Chlorobi species BMC Evol Biol 2007 7 71 10.1186/1471-2148-7-71 17488508 PMC1887533

[B27] Gupta RS Mok A Phylogenomics and signature proteins for the alpha Proteobacteria and its main groups BMC Microbiol 2007 7 106 10.1186/1471-2180-7-106 18045498 PMC2241609

[B28] Dutilh BE Snel B Ettema TJ Huynen MA Signature genes as a phylogenomic tool Mol Biol Evol 2008 25 1659 1667 10.1093/molbev/msn115 18492663 PMC2464742

[B29] Martin KA Siefert JL Yerrapragada S Lu Y McNeill TZ Moreno PA Weinstock GM Widger WR Fox GE Cyanobacterial signatures genes Photosynth Res 2003 75 211 221 10.1023/A:1023990402346 16228602

[B30] Mulkidjanian AY Koonin EV Makarova KS Mekhedov SL Sorokin A Wolf YI Dufresne A Partensky F Burd H Kaznadzey D Haselkorn R Galperin MY The cyanobacterial genome core and the origin of photosynthesis Proc Natl Acad Sci USA 2006 103 13126 13131 10.1073/pnas.0605709103 16924101 PMC1551899

[B31] Ciccarelli FD Doerks T von Mering C Creevey CJ Snel B Bork P Toward automatic reconstruction of a highly resolved tree of life Science 2006 311 1283 1287 10.1126/science.1123061 16513982

[B32] Gogarten JP Townsend JP Horizontal gene transfer, genome innovation and evolution Nat Rev Microbiol 2005 3 679 687 10.1038/nrmicro1204 16138096

[B33] Zhaxybayeva O Doolittle WF Papke RT Gogarten JP Intertwined evolutionary histories of marine Synechococcus and Prochlorococcus marinus Genome Biology and Evolution 2009 1 325 339 10.1093/gbe/evp032 PMC2817427 20333202

[B34] Herbeck JT Degnan PH Wernegreen JJ Nonhomogeneous model of sequence evolution indicates independent origins of primary endosymbionts within the enterobacteriales (gamma-Proteobacteria) Mol Biol Evol 2005 22 520 532 10.1093/molbev/msi036 15525700

[B35] Harris JK Kelley ST Spiegelman GB Pace NR The genetic core of the universal ancestor Genome Res 2003 13 407 412 10.1101/gr.652803 12618371 PMC430263

[B36] Nakamura Y Kaneko T Sato S Mimuro M Miyashita H Tsuchiya T Sasamoto S Watanabe A Kawashima K Kishida Y Kiyokawa C Kohara M Matsumoto M Matsuno A Nakazaki N Shimpo S Takeuchi C Yamada M Tabata S Complete genome structure of Gloeobacter violaceus PCC a cyanobacterium that lacks thylakoids DNA Res 7421 10 137 145 10.1093/dnares/10.4.137 14621292

[B37] Honda D Yokota A Sugiyama J Detection of seven major evolutionary lineages in cyanobacteria based on the 16S rRNA gene sequence analysis with new sequences of five marine *Synechococcus *strains J Mol Evol 1999 48 723 739 10.1007/PL00006517 10229577

[B38] Giovannoni SJ Turner S Olsen GJ Barns S Lane DJ Pace NR Evolutionary relationships among cyanobacteria and green chloroplasts J Bacteriol 1988 170 3584 3592 3136142 10.1128/jb.170.8.3584-3592.1988 PMC211332

[B39] Seo PS Yokota A The phylogenetic relationships of cyanobacteria inferred from 16S rRNA, gyrB, rpoC1 and rpoD1 gene sequences J Gen Appl Microbiol 2003 49 191 203 10.2323/jgam.49.191 12949700

[B40] Rocap G Larimer FW Lamerdin J Malfatti S Chain P Ahlgren NA Arellano A Coleman M Hauser L Hess WR Johnson ZI Land M Lindell D Post AF Regala W Shah M Shaw SL Steglich C Sullivan MB Ting CS Tolonen A Webb EA Zinser ER Chisholm SW Genome divergence in two Prochlorococcus ecotypes reflects oceanic niche differentiation Nature 2003 424 1042 1047 10.1038/nature01947 12917642

[B41] Rocap G Distel DL Waterbury JB Chisholm SW Resolution of Prochlorococcus and Synechococcus ecotypes by using 16S-23S ribosomal DNA internal transcribed spacer sequences Appl Environ Microbiol 2002 68 1180 1191 10.1128/AEM.68.3.1180-1191.2002 11872466 PMC123739

[B42] Gao B Mohan R Gupta RS Phylogenomics and protein signatures elucidating the evolutionary relationships among the Gammaproteobacteria Int J Syst Evol Microbiol 2009 59 234 247 10.1099/ijs.0.002741-0 19196760

[B43] Gao B Gupta RS Phylogenomic analysis of proteins that are distinctive of *Archaea *and its main subgroups and the origin of methanogenesis BMC Genomics 2007 8 86 10.1186/1471-2164-8-86 17394648 PMC1852104

[B44] Lerat E Daubin V Ochman H Moran NA Evolutionary Origins of Genomic Repertoires in Bacteria PLoS Biol 2005 3 e130 10.1371/journal.pbio.0030130 15799709 PMC1073693

[B45] Swingley WD Chen M Cheung PC Conrad AL Dejesa LC Hao J Honchak BM Karbach LE Kurdoglu A Lahiri S Mastrian SD Miyashita H Page L Ramakrishna P Satoh S Sattley WM Shimada Y Taylor HL Tomo T Tsuchiya T Wang ZT Raymond J Mimuro M Blankenship RE Touchman JW Niche adaptation and genome expansion in the chlorophyll d-producing cyanobacterium Acaryochloris marina Proc Natl Acad Sci USA 2008 105 2005 2010 10.1073/pnas.0709772105 18252824 PMC2538872

[B46] Nakamura Y Kaneko T Sato S Ikeuchi M Katoh H Sasamoto S Watanabe A Iriguchi M Kawashima K Kimura T Kishida Y Kiyokawa C Kohara M Matsumoto M Matsuno A Nakazaki N Shimpo S Sugimoto M Takeuchi C Yamada M Tabata S Complete genome structure of the thermophilic cyanobacterium *Thermosynechococcus elongatus BP-1* DNA Research 2002 9 123 130 10.1093/dnares/9.4.123 12240834

[B47] Turner S Pryer KM Miao VP Palmer JD Investigating deep phylogenetic relationships among cyanobacteria and plastids by small subunit rRNA sequence analysis J Eukaryot Microbiol 1999 46 327 338 10.1111/j.1550-7408.1999.tb04612.x 10461381

[B48] Kaneko T Nakamura Y Wolk CP Kuritz T Sasamoto S Watanabe A Iriguchi M Ishikawa A Kawashima K Kimura T Kishida Y Kohara M Matsumoto M Matsuno A Muraki A Nakazaki N Shimpo S Sugimoto M Takazawa M Yamada M Yasuda M Tabata S Complete genomic sequence of the filamentous nitrogen-fixing cyanobacterium *Anabaena sp*. strain PCC 7120 DNA Res 2001 8 205 213 10.1093/dnares/8.5.205 11759840

[B49] Caspi J Amitai G Belenkiy O Pietrokovski S Distribution of split DnaE inteins in cyanobacteria Mol Microbiol 2003 50 1569 1577 10.1046/j.1365-2958.2003.03825.x 14651639 PMC7168405

[B50] Adams DG Heterocyst formation in cyanobacteria Curr Opin Microbiol 2000 3 618 624 10.1016/S1369-5274(00)00150-8 11121783

[B51] Dufresne A Salanoubat M Partensky F Artiguenave F Axmann IM Barbe V Duprat S Galperin MY Koonin EV Le Gall F Makarova KS Ostrowski M Oztas S Robert C Rogozin IB Scanlan DJ De Marsac NT Weissenbach J Wincker P Wolf YI Hess WR Genome sequence of the cyanobacterium *Prochlorococcus marinus *SS120, a nearly minimal oxyphototrophic genome Proc Natl Acad Sci USA 2003 100 10020 10025 10.1073/pnas.1733211100 12917486 PMC187748

[B52] Palenik B Brahamsha B Larimer FW Land M Hauser L Chain P Lamerdin J Regala W Allen EE McCarren J Paulsen I Dufresne A Partensky F Webb EA Waterbury J The genome of a motile marine Synechococcus Nature 2003 424 1037 1042 10.1038/nature01943 12917641

[B53] Palenik B Ren Q Dupont CL Myers GS Heidelberg JF Badger JH Madupu R Nelson WC Brinkac LM Dodson RJ Durkin AS Daugherty SC Sullivan SA Khouri H Mohamoud Y Halpin R Paulsen IT Genome sequence of Synechococcus CC9311: Insights into adaptation to a coastal environment Proc Natl Acad Sci USA 2006 103 13555 13559 10.1073/pnas.0602963103 16938853 PMC1569201

[B54] Sugishima M Migita CT Zhang X Yoshida T Fukuyama K Crystal structure of heme oxygenase-1 from cyanobacterium Synechocystis sp. PCC 6803 in complex with heme Eur J Biochem 2004 271 4517 4525 10.1111/j.1432-1033.2004.04411.x 15560792

[B55] Heyes DJ Scrutton NS Conformational changes in the catalytic cycle of protochlorophyllide oxidoreductase: what lessons can be learnt from dihydrofolate reductase? Biochem Soc Trans 2009 37 354 357 10.1042/BST0370354 19290861

[B56] Moore LR Rocap G Chisholm SW Physiology and molecular phylogeny of coexisting Prochlorococcus ecotypes Nature 1998 393 464 467 10.1038/30965 9624000

[B57] Ferris MJ Palenik B Niche adaptation in ocean cyanobacteria Nature 1998 396 226 228 10.1038/24297

[B58] Narra HP Cordes MH Ochman H Structural features and the persistence of acquired proteins Proteomics 2008 8 4772 4781 10.1002/pmic.200800061 18924109 PMC3014317

[B59] Gao B Mohan R Gupta RS Phylogenomics and protein signatures elucidating the evolutionary relationships among the *Gammaproteobacteria* Int J Syst Evol Microbiol 2009 59 234 247 10.1099/ijs.0.002741-0 19196760

[B60] Siew N Fischer D Analysis of singleton ORFans in fully sequenced microbial genomes Proteins 2003 53 241 251 10.1002/prot.10423 14517975

[B61] Kuo CH Ochman H The fate of new bacterial genes FEMS Microbiol Rev 2009 33 38 43 10.1111/j.1574-6976.2008.00140.x 19054121

[B62] Fang G Rocha EP Danchin A Persistence drives gene clustering in bacterial genomes BMC Genomics 2008 9 4 10.1186/1471-2164-9-4 18179692 PMC2234087

[B63] Gupta RS Molecular signatures (unique proteins and conserved Indels) that are specific for the epsilon proteobacteria (Campylobacterales) BMC Genomics 2006 7 167 10.1186/1471-2164-7-167 16817973 PMC1557499

[B64] Gupta RS Griffiths E Chlamydiae-specific proteins and indels: novel tools for studies Trends Microbiol 2006 14 527 535 10.1016/j.tim.2006.10.002 17049238

[B65] Gogarten JP Doolittle WF Lawrence JG Prokaryotic evolution in light of gene transfer Mol Biol Evol 2002 19 2226 2238 12446813 10.1093/oxfordjournals.molbev.a004046

[B66] Raymond J Zhaxybayeva O Gogarten JP Gerdes SY Blankenship RE Whole-genome analysis of photosynthetic prokaryotes Science 2002 298 1616 1620 10.1126/science.1075558 12446909

[B67] Rokas A Holland PW Rare genomic changes as a tool for phylogenetics Trends Ecol Evol 2000 15 454 459 10.1016/S0169-5347(00)01967-4 11050348

[B68] Huang J Gogarten JP Ancient gene transfer as a tool in phylogenetic reconstruction Methods Mol Biol 2009 532 127 139 full_text 19271182 10.1007/978-1-60327-853-9_7

[B69] von Mering C Hugenholtz P Raes J Tringe SG Doerks T Jensen LJ Ward N Bork P Quantitative phylogenetic assessment of microbial communities in diverse environments Science 2007 315 1126 1130 10.1126/science.1133420 17272687

[B70] Doerks T von Mering C Bork P Functional clues for hypothetical proteins based on genomic context analysis in prokaryotes Nucleic Acids Res 2004 32 6321 6326 10.1093/nar/gkh973 15576358 PMC535681

[B71] Fang G Rocha E Danchin A How essential are nonessential genes? Mol Biol Evol 2005 22 2147 2156 10.1093/molbev/msi211 16014871

[B72] Yang Z The power of phylogenetic comparison in revealing protein function Proc Natl Acad Sci USA 2005 102 3179 3180 10.1073/pnas.0500371102 15728394 PMC552944

[B73] Singh B Gupta RS Conserved inserts in the Hsp60 (GroEL) and Hsp70 (DnaK) proteins are essential for cellular growth Mol Genet Genomics 2009 281 361 373 10.1007/s00438-008-0417-3 19127371

[B74] Roberts RJ Identifying protein function--a call for community action PLoS Biol 2004 2 E42 10.1371/journal.pbio.0020042 15024411 PMC368155

[B75] Galperin MY Koonin EV 'Conserved hypothetical' proteins: prioritization of targets for experimental study Nucleic Acids Res 2004 32 5452 5463 10.1093/nar/gkh885 15479782 PMC524295

[B76] Danchin A From protein sequence to function Curr Opin Struct Biol 1999 9 363 367 10.1016/S0959-440X(99)80049-9 10408894

[B77] Jeanmougin F Thompson JD Gouy M Higgins DG Gibson TJ Multiple sequence alignment with Clustal x Trends Biochem Sci 1998 23 403 405 10.1016/S0968-0004(98)01285-7 9810230

[B78] Castresana J Selection of conserved blocks from multiple alignments for their use in phylogenetic analysis Mol Biol Evol 2000 17 540 552 10742046 10.1093/oxfordjournals.molbev.a026334

[B79] Kimura M The Neutral Theory of Molecular Evolution 1983 Cambridge: Cambridge University Press

[B80] Peer Y Van de De Wachter R TREECON for Windows: a software package for the construction and drawing of evolutionary trees for the Microsoft Windows environment Comput Appl Biosci 1994 10 569 570 7828077 10.1093/bioinformatics/10.5.569

[B81] Schmidt HA Strimmer K Vingron M von Haeseler A TREE-PUZZLE: maximum likelihood phylogenetic analysis using quartets and parallel computing Bioinformatics 2002 18 502 504 10.1093/bioinformatics/18.3.502 11934758

[B82] Altschul SF Madden TL Schaffer AA Zhang J Zhang Z Miller W Lipman DJ Gapped BLAST and PSI-BLAST: a new generation of protein databases search programs Nucleic Acids Research 1997 25 3389 3402 10.1093/nar/25.17.3389 9254694 PMC146917

[B83] Sugita C Ogata K Shikata M Jikuya H Takano J Furumichi M Kanehisa M Omata T Sugiura M Sugita M Complete nucleotide sequence of the freshwater unicellular cyanobacterium Synechococcus elongatus PCC 6301 chromosome: gene content and organization Photosynth Res 2007 93 55 67 10.1007/s11120-006-9122-4 17211581

[B84] Dufresne A Ostrowski M Scanlan DJ Garczarek L Mazard S Palenik BP Paulsen IT De Marsac NT Wincker P Dossat C Ferriera S Johnson J Post AF Hess WR Partensky F Unraveling the genomic mosaic of a ubiquitous genus of marine cyanobacteria Genome Biol 2008 9 R90 10.1186/gb-2008-9-5-r90 18507822 PMC2441476

[B85] Kaneko T Sato S Kotani H Tanaka A Asamizu E Nakamura Y Miyajima N Hirosawa M Sugiura M Sasamoto S Kimura T Hosouchi T Matsuno A Muraki A Nakazaki N Naruo K Okumura S Shimpo S Takeuchi C Wada T Watanabe A Yamada M Yasuda M Tabata S Sequence analysis of the genome of the unicellular cyanobacterium *Synechocystis sp*. strain PCC6803. II. Sequence determination of the entire genome and assignment of potential protein-coding regions DNA Research 1996 3 109 136 10.1093/dnares/3.3.109 8905231

