# Supplementary material for: Signature proteins for the major clades of Cyanobacteria
Source: BMC Evol Biol. 2010 Jan 25;10:24. doi: 10.1186/1471-2148-10-24 (PMC2823733; doi:10.1186/1471-2148-10-24)
Supplement: Additional file 1 — List of proteins used in phylogenetic analyses. The information for various proteins regarding their lengths, accession numbers, Gene bank IDs, locus tag for Nostoc sp. PCC7120 and COG groups is provided. [file 1471-2148-10-24-S1.PDF]

Additional file 1

List of Proteins Used in Phylogenetic Analyses

Protein Name	Length	Gene Bank ID No.	locus tag	COG
Elongation factor G	692	17231830	all4338	COG0480J
DNA gyrase subunit A	872	17228355	all0860	COG0188L
DNA gyraseB	645	17232757	all5265	COG0187L
Isoleucyl-tRNA synthetase	960	17228568	alr1073	COG0060J
DNA-directed RNA polymerase alpha subunit	315	17231683	all4191	COG0202K
DNA-directed RNA polymerase beta subunit	1131	17229086	alr1594	COG0085K
DNA-directed RNA polymerase beta’ subunit	625	17229087	alr1595	COG0086K
Preprotein translocase SecY subunit	437	17231689	all4197	COG0201U
Seryl-tRNA synthetase	426	17231468	all3976	COG0172J
Valyl-tRNA synthetase	1014	17228813	all1318	COG0525J
Phosphatidate cytidylyltransferase	294	17231367	all3875	COG0575I
Elongation factor P	185	17232550	all5058	COG0231J
Signal recognition particle protein	490	17229444	alr1952	COG0541U
Serine hydroxymethyltransferase	427	17232298	alr4806	COG0112E
DNA polymerase III subunit delta'	329	17230936	alr3444	COG1466L
Translation initiation factor IF-2	1039	17231324	alr3832	COG0532J
Dimethyladenosine transferase	271	17230721	alr3229	COG0030J
Leucyl-tRNA synthetase	872	17230775	alr3283	COG0495J
Methionyl-tRNA synthetase	530	17227729	all0233	COG0143J
Phenylalanyl-tRNA synthetase	330	17232337	all4845	COG0016J
DNA polymerase I	977	17228749	alr1254	COG0749L
Prolyl-tRNA synthetase	604	17232545	alr5053	COG0442J
Recombinase A	357	17230764	all3272	COG0468
50S ribosomal protein L15	148	17231690	all4198	COG0200J
50S ribosomal protein L2	287	17231704	all4212	COG0090J
50S ribosomal protein L4	210	17231706	all4214	COG0088J
50S ribosomal protein L5	182	17231695	all4203	COG0094J
50S ribosomal protein L6	182	17231693	all4201	COG0097J
30S ribosomal protein S2	265	17232284	all4792	COG0052J
30S ribosomal protein S3	260	17231701	all4209	COG0092J
30S ribosomal protein S8	133	17231694	all4202	COG0096J
30S ribosomal protein S11	131	17231684	all4192	COG0100J
30S ribosomal protein S12	127	17231832	all4340	COG0048J
30S ribosomal protein S13	126	17231685	all4193	COG0099J
30S ribosomal protein S15	89	17228244	as10749	COG0184J
30S ribosomal protein S17	81	17231698	asl4206	COG0186J
30S ribosomal protein S19	92	17231703	asl4211	COG0185J
30S ribosomal protein S5	174	17231691	all4199	COG0098J
Preprotein translocase subunit	930	17232343	alr4851	COG0653U
Tryptophanyl-tRNA synthetase	335	17228764	all1269	COG0180J
Tyrosyl-tRNA synthetase	398	17230474	alr2982	COG0162J
Translation-associated GTPase	363	17228218	all0723	COG0012J
Cell division protein	546	17229251	all1759	COG0552U
DNA helicase II	772	17231987	all4495	CO0210L

Note: Accession number and other nformation provided in this Table is for the Nostoc sp. PCC 7120 homologs.
