# Supplementary material for: Signature proteins for the major clades of Cyanobacteria
Source: BMC Evol Biol. 2010 Jan 25;10:24. doi: 10.1186/1471-2148-10-24 (PMC2823733; doi:10.1186/1471-2148-10-24)

Additional file 2

A neighbor-joining tree for sequenced cyanobacteria based on concatenated sequences for 44 conserved proteins. The tree was rooted using sequences for *B. subtilis* and *S. aureus*.

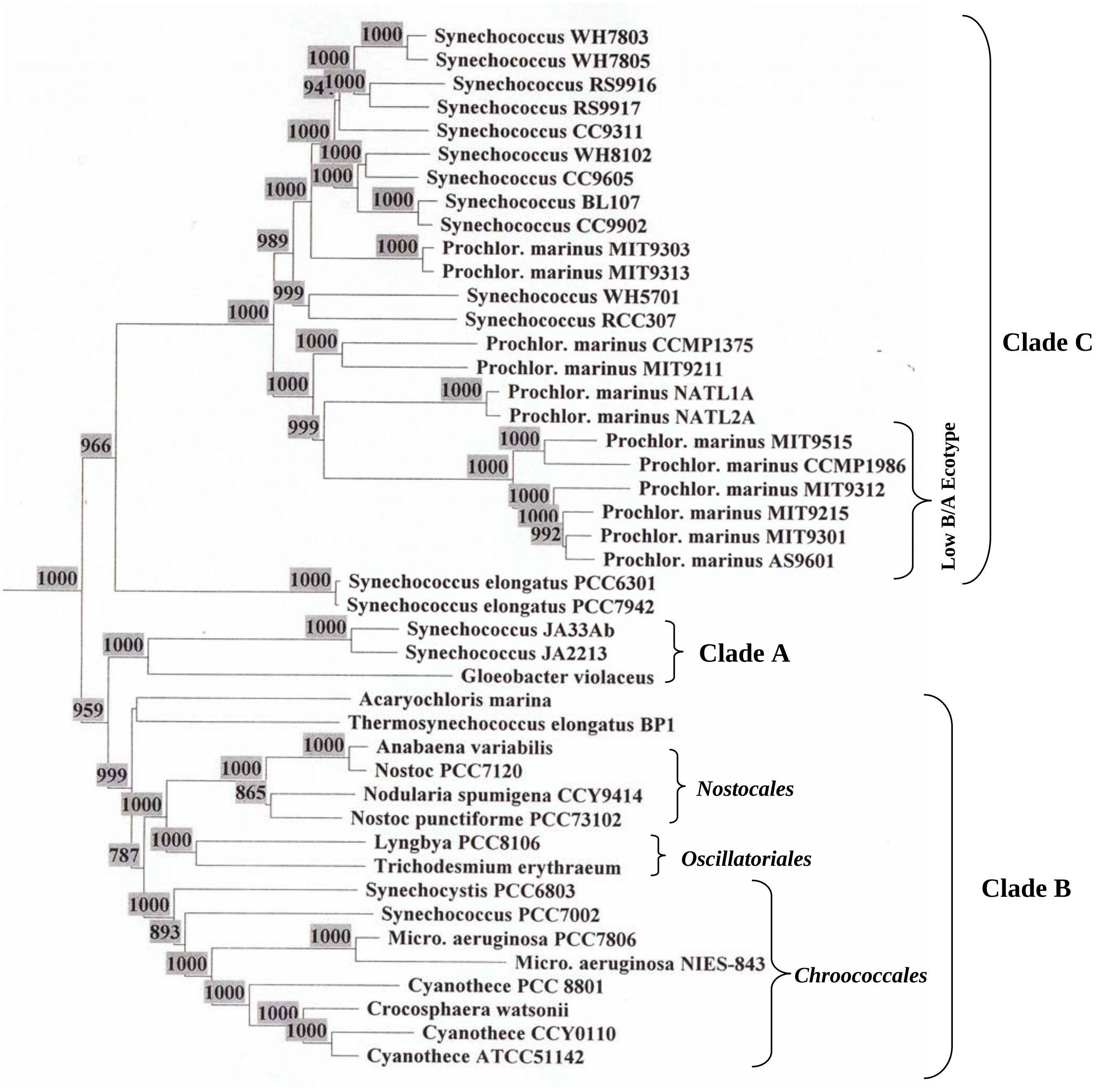

Supplement: Additional file 2 — Neighbour-joining tree for the sequenced Cyanobacteria. A neighbour-joining, bootstrapped tree for 44 cyanobacteria based on concatenated sequences for 44 proteins listed in additional file 1. The sequences for B. subtilis and S. aureus were used to root this tree. [file 1471-2148-10-24-S2.PDF]
