# Supplementary material for: Signature proteins for the major clades of Cyanobacteria
Source: BMC Evol Biol. 2010 Jan 25;10:24. doi: 10.1186/1471-2148-10-24 (PMC2823733; doi:10.1186/1471-2148-10-24)
Supplement: Additional file 3 — Proteins that are specific for the Clade A of Cyanobacteria.. All of the proteins listed in this Table are specific for Clade A, which consists of G. violaceus and Synechococcus sps. JA-3-3Ab and JA-2-3B'a. [file 1471-2148-10-24-S3.PDF]

Additional file 3

Proteins Specific for Clade A Cyanobacteria

**Protein**	**Function (Length)**	**Protein**	**Function (Length)**
YP_473534/CYA_0039	Hypothetical (488)	YP_474887/CYA_1456	Hypothetical (345)
YP_473576/CYA_0083	Hypothetical (273)	YP_475160/CYA_1742	Hypothetical (127)
YP_473749/CYA_0265	Hypothetical (193)	YP_475218/CYA_1802	Hypothetical (295)
YP_474072/CYA_0593	Hypothetical (101)	YP_475362/CYA_1954	Putative lipoprotein (191)
YP_474248/CYA_0778	Hypothetical (73)	YP_475414/CYA_2008	Hypothetical (139)
YP_474437/CYA_0974	Hypothetical (310)	YP_475567/CYA_2164	Hypothetical (410)
YP_474812/CYA_1378	Hypothetical (103)	YP_475614/CYA_212	Hypothetical (368)

Note: Clade A is comprised of *G. violaceus, Synechococcus sp. JA-3-3Ab and Synechococcus sp. JA-2-3B'a*
