# Supplementary material for: Signature proteins for the major clades of Cyanobacteria
Source: BMC Evol Biol. 2010 Jan 25;10:24. doi: 10.1186/1471-2148-10-24 (PMC2823733; doi:10.1186/1471-2148-10-24)
Supplement: Additional file 4 — Proteins specific for the Nostocales, Oscillatoriales and Chroococcales orders.. As above [file 1471-2148-10-24-S4.PDF]

Additional file 4

Proteins Specific for Nostocales, Oscillatoriales and Chroococcales[+]

Proteins specific for these groups but missing in 1-2 species			
**Protein**	**Function (length)**	**Protein**	**Function (length)**
BAA17401/slr2070	Hypothetical (284)	NP_487024/ alr2984	Hypothetical (238)
NP_484540/all0496	Hypothetical (242)	NP_487041/ asr3001	Hypothetical (55)
NP_484143/alr0099	Hypothetical (401)	NP_487243/ alr3203	Hypothetical (162)
NP_484246/alr0202	Hypothetical (404)	NP_488349/alr4309	Hypothetical (153)
NP_485295/all1252	Hypothetical (171)	NP_488474/all4434	Hypothetical (116)
NP_485769/all1729	Hypothetical (214)	NP_488482/all4442	Hypothetical (552)
NP_487625/all3585	Hypothetical (278)	NP_488485/all4445 [a]	Hypothetical (166)
NP_485014/alr0971	Hypothetical (570)	NP_488493/all4453[#]	Hypothetical (186)
NP_486614/alr2574	Hypothetical (292)	NP_488783/asl4743	Hypothetical (63)
NP_488005/alr3965	Hypothetical (285)	NP_488950/asr4910	Hypothetical (59)
NP_486550/ alr2510	Hypothetical (316)	NP_489369/alr5329	Hypothetical (211)
**Proteins specific for these groups but missing in 3 or more species**			
BAD01882/slr5112	unknown protein (377)	NP_486564/ alr2524	Hypothetical (103)
NP_484560/asr0516	Hypothetical (56)	NP_486567/ alr2527	Hypothetical (123)
NP_484934/all0891	Hypothetical (140)	NP_487753/all3713	Hypothetical (266)
NP_485467/all1424	Hypothetical (124)	NP_489311/alr5271	Hypothetical (188)
NP_486563/ asr2523	Hypothetical (91)		

[+] Proteins that are present in most of the Nostocales, Oscillatoriales and Chroococcales are listed in Table 2.

[#] also found in one of the clade A cyanobacteria

[a] homolog showing significant similarity also present in *Methanospirillum hungtaei*
