# Supplementary material for: Signature proteins for the major clades of Cyanobacteria
Source: BMC Evol Biol. 2010 Jan 25;10:24. doi: 10.1186/1471-2148-10-24 (PMC2823733; doi:10.1186/1471-2148-10-24)
Supplement: Additional file 5 — Proteins specific for the Nostocales order.. As above [file 1471-2148-10-24-S5.PDF]

Additional file 5

Proteins that are specific for Nostocales[+]

**Protein**	**Function (length)**	**Protein**	**Function (length)**
NP_485191/alr1148	Hypothetical (364)	NP_488605/asl4565	
NP_485277/asr1234	Hypothetical (98)	NP_488803/ all4763	Hypothetical (207)
NP_485332/asr1289	Hypothetical (70)	NP_488822/ all4782	Hypothetical (124)
NP_485438/all1395	Hypothetical (140)	NP_488828/alr4788	Hypothetical (173))
NP_485455/asl1412	Hypothetical (91)	NP_488852/ alr4812	heterocyst differentiation related (215)
NP_485789/asl1749	Hypothetical (90)	NP_488867/ asl4827	GDP-D-mannose dehydratase(344)
NP_485813/asr1775	Hypothetical (88)	NP_489002/ all4962	Hypothetical (420)
NP_485854/all1814	Hypothetical (189)	NP_489027/asr4987	Hypothetical (74)
NP_485914/alr1874	Hypothetical (191)	NP_489045/alr5005	Hypothetical (153)
NP_485973/ asl1933	Hypothetical (77)	NP_489111/asr5071	Hypothetical (95)
NP_485974/ asl1934	Hypothetical (77)	NP_489329/asr5289	Hypothetical (53)
NP_485975/ asl1935	Hypothetical (77)	NP_486139/all2099	Hypothetical (256)
NP_484139/asl0095	Hypothetical (69)	NP_486953/ all2913	Hypothetical (261)
NP_489183/alr5143	Hypothetical (116)		
**Proteins specific to Nostocales but missing in one to two species**			
NP_484250/asl0206	Hypothetical (60)	NP_486399/alr2359	Hypothetical (135)
NP_484357/all0313	Hypothetical (110)	NP_486410/asl2370	Hypothetical(63)
NP_484478/all0434	Hypothetical (110)	NP_486599/alr2559	Hypothetical (306)
NP_484767/asl0724	Hypothetical (69)	NP_486600/alr2560	Hypothetical (319)
NP_484860/asl0817	Hypothetical(66)	NP_486640/asr2600	Hypothetical(62)
NP_484933/asr0890	Hypothetical (75)	NP_486744/all2704	Hypothetical (310)
NP_485081/asr1038	Hypothetical (80)	NP_486767/asl2727	Hypothetical (59)
NP_485098/alr1055	Hypothetical (181)	NP_486775/alr2735	Hypothetical (210)
NP_485099/alr1056	Hypothetical (151)	NP_486794/all2754	Hypothetical (179)
NP_485190/alr1147	Hypothetical (1128)	NP_486908/ all2868	Hypothetical (231)
NP_485238/asr1195	Hypothetical (90)	NP_487062/ all3022	Hypothetical (153)
NP_485311/all1268	Hypothetical (131)	NP_487174/ asr3134	Hypothetical (69)
NP_485343/alr1300	Hypothetical (150)	NP_487182/alr3142	hypotherical(168)
NP_485344/alr1301	Hypothetical (219)	NP_487377/ alr3337	Hypothetical (115)
NP_485423/all1380	Hypothetical (192)	NP_487405/ alr3365	Hypothetical (332)
NP_485612/asr1572	Hypothetical (52)	NP_487521/ alr3481	Hypothetical (185)
NP_485692/alr1652	Hypothetical (259)	NP_487522/ alr3482	Hypothetical (110)
NP_485693/alr1653	Hypothetical (315)	NP_487599/ all3559	Hypothetical (280)
NP_485694/alr1654	Hypothetical (327)	NP_487641/alr3601	Hypothetical (498)
NP_485765/all1725	Hypothetical (136)	NP_487773/ all3733	Hypothetical(105)
NP_485774/asr1734	Hypothetical (93)	NP_487792/alr3752	Hypothetical (183)
NP_485830/alr1790	Hypothetical (113)	NP_488602/asl4562	Hypothetical (70)
NP_485855/alr1815	Hypothetical (212)	NP_488960 /asr4920	Hypothetical (80)
NP_485870/all1830	Hypothetical(102)	NP_489020/ all4980	Hypothetical (131)
NP_485947/asr1907	Hypothetical(55)	NP_489180/ alr5140	Hypothetical (103)
NP_485978/ asl1938	Hypothetical (85)	NP_489181/ alr5141	Hypothetical (110)
NP_486056/asr2016	Hypothetical(97)	NP_489182/asr5142	Hypothetical (80)
NP_486129/all2089	Hypothetical (160)	NP_489353/asr5313	Hypothetical (71)
NP_486339/asl2299	Hypothetical(74)	NP_489409/all5369	Hypothetical (160)

[+] Many proteins that are specific for Nostocales are listed in Table 4
