# Supplementary material for: Signature proteins for the major clades of Cyanobacteria
Source: BMC Evol Biol. 2010 Jan 25;10:24. doi: 10.1186/1471-2148-10-24 (PMC2823733; doi:10.1186/1471-2148-10-24)
Supplement: Additional file 6 — Clade C proteins showing anomalous behavior of Pro. marinus MIT9303 and MIT9313.. This table describes two sets of proteins: (a) Proteins that are specific for Clade C Synechococcus strains/isolates that are also found in Pro. marinus MIT9303 and MIT9313 and (b) Proteins specific for various other Prochlorococcus marinus strains/isolates, but which are missing in Pro. marinus MIT9303 and MIT9313. [file 1471-2148-10-24-S6.PDF]

Additional file 5

Clade C Proteins showing anomalous behavior of *Pro. marinus MIT9303* and *MIT9313*

**(a) Proteins Specific for Clade C Synechococcus species/strains that are also found in *Pro. marinus MIT9303* and *MIT9313***			
**Protein**	**Function (Length)**	**Protein**	**Function (Length)**
NP_896150/SYNW0055	Hypothetical (183)	NP_897350/SYNW1257*	Hypothetical (257)
NP_896317/SYNW0222	Hypothetical (170)	NP_897419/SYNW1326*	Hypothetical(88)
NP_896619/SYNW0524*	Hypothetical (94)	NP_897605/SYNW1512*	Hypothetical (97)
NP_896825/SYNW0732	Hypothetical (81)	NP_897764/SYNW1673*	Hypothetical(116)
NP_896954l/SYNW0861*	Hypothetical(320)	NP_898124/SYNW2033*	Hypothetical (160)
NP_897005/SYNW0912	Hypothetical (103)	NP_898243/SYNW2152	Hypothetical (140)
NP_897065/SYNW0972*	Hypothetical (347)	NP_898246/SYNW2155	Hypothetical (98)
NP_897066/SYNW0973	Hypothetical (440)	NP_898250/SYNW2159	Hypothetical (189)
NP_897133/SYNW1040*	Hypothetical (85)	NP_898302/SYNW2211	Hypothetical (162)
NP_897226/SYNW1133*	Hypothetical(118)	NP_898379/SYNW2290	Hypothetical (184)
NP_897244/SYNW1151	Hypothetical (83)	NP_898474/SYNW2385	Hypothetical (164)
**(b) Proteins Specific for *Prochlorococcus* but Missing in *Pro. marinus MIT9303* and *MIT9313***			
YP_001091594	hypothetical (81)	YP_001484295*	hypothetical (167)
YP_001483242*	hypothetical (108)	YP_001484304	hypothetical (77)
YP_001483272*	hypothetical (51)	YP_001484324	hypothetical (79)
YP_001483349	possible Signal peptide (119)	YP_001484334	hypothetical (65)
YP_001483576	hypothetical (105)	YP_001484370	hypothetical (92)
YP_001483586*	Spectrin repeat-containing (78)	YP_001484378*	hypothetical (117)
YP_001483654	hypothetical (63)	YP_001484525*	hypothetical (121)
YP_001483909	hypothetical (77)	YP_001484830*	hypothetical (109)
YP_001483985	hypothetical (94)	YP_001483987*	hypothetical (56)
YP_001484003	hypothetical (127)	YP_001483907	hypothetical (94)
YP_001483989	hypothetical (69)	YP_001483991*	hypothetical (68)
YP_001483976	hypothetical (45)	YP_001484030*	hypothetical (116)
YP_001484114	hypothetical (37)	YP_001484106*	hypothetical (113)
YP_001484291	hypothetical (63)	YP_001484228*	hypothetical (207)
YP_001484293*	hypothetical (73)	YP_001484238*	hypothetical (58)
YP_00148359*	hypothetical (65)	YP_001484288*	hypothetical (75)
YP_001483606*	possible phosphoenolpyruvate (125)	YP_001484359*	hypothetical (47)
YP_001483616*	hypothetical (65)	YP_001484361	hypothetical (48)
YP_001483984*	hypothetical (65)	YP_001484028	hypothetical (60)
YP_001484331	hypothetical (78)		

* - Missing in 1-2 isolates
