# Supplementary material for: Signature proteins for the major clades of Cyanobacteria
Source: BMC Evol Biol. 2010 Jan 25;10:24. doi: 10.1186/1471-2148-10-24 (PMC2823733; doi:10.1186/1471-2148-10-24)
Supplement: Additional file 7 — Proteins specific for the Low B/A ecotype Pro. marinus strains/isolates. [file 1471-2148-10-24-S7.PDF]

Additional file 7

Proteins specific to Low B/A Ecotype *Prochlorococcus* Clade

Protein	Function (length)	Protein	Function (length)
YP_001483315	hypothetical (113)	YP_001484344	hypothetical (46)
YP_001483342	hypothetical (89)	YP_001484352	hypothetical (124)
YP_001483385	hypothetical (486)	YP_001484354	hypothetical (79)
YP_001483434	hypothetical (105)	YP_001484379	hypothetical (63)
YP_001483571	hypothetical (67)	YP_001484437	hypothetical (50)
YP_001483594	hypothetical (59)	YP_001484459	hypothetical (54)
YP_001483604	hypothetical (47)	YP_001484488	hypothetical (75)
YP_001483623	hypothetical (61)	YP_001484491	hypothetical (54)
YP_001483702	hypothetical (67)	YP_001484519	hypothetical (44)
YP_001483718	hypothetical (161)	YP_001484532	hypothetical (53)
YP_001483732	hypothetical (129)	YP_001484533	hypothetical (60)
YP_001483944	hypothetical (49)	YP_001484535	hypothetical (86)
YP_001483977	hypothetical (49)	YP_001484538	hypothetical (71)
YP_001483994	hypothetical (84)	YP_001484539	hypothetical (83)
YP_001483995	possible D12 class N6 (75)	YP_001484581	hypothetical (56)
YP_001483997	possible DnaJ central domain (83)	YP_001484747	hypothetical (80)
YP_001484005	hypothetical (45)	YP_001484748	hypothetical (103)
YP_001484020	hypothetical (137)	YP_001484787	hypothetical (57)
YP_001484021	hypothetical (93)	YP_001484792	hypothetical (88)
YP_001484050	possible Adenylate cyclase (384)	YP_001484801	hypothetical (78)
YP_001484112	hypothetical (72)	YP_001484808	hypothetical (60)
YP_001484157	hypothetical (110)	YP_001484809	hypothetical (42)
YP_001484240	hypothetical (58)	YP_001484811	possible Type I restriction modification DNA s (117)
YP_001484242	influenza RNA-dependent RNA polymerase-like protein (73)	YP_001484812	hypothetical (51)
YP_001484246	hypothetical (250)	YP_001484814	hypothetical (50)
YP_001484265	hypothetical (122)	YP_001484816	hypothetical (39)
YP_001484294	hypothetical (74)	YP_001484824	hypothetical (47)
YP_001484296	hypothetical (46)	YP_001484826	hypothetical (158)
YP_001484308	hypothetical (107)	YP_001484829	hypothetical (36)
YP_001484309	hypothetical (49)	YP_001484881	hypothetical (34)
YP_001484321	possible Photosystem II (53)	YP_001484908	hypothetical (52)
YP_001484322	hypothetical (61)	YP_001484983	hypothetical (70)
YP_001484339	possible Virion host shutoff (97)	YP_001485104	hypothetical (109)
YP_001484343	hypothetical (59)		
**Missing in 1 or 2 species**			
YP_001091778	hypothetical (42)	YP_001484239	hypothetical (57)
YP_001483252	hypothetical (47)	YP_001484313	hypothetical (63)
YP_001483268	hypothetical (45)	YP_001484314	hypothetical (68)
YP_001483333	hypothetical (60)	YP_001484316	hypothetical (61)
YP_001483438	hypothetical (51)		
YP_001483563	hypothetical (45)	YP_001484345	possible Integrin alpha cytoplasmic region (100)

YP_001483569	hypothetical (68)	YP_001484346	hypothetical (61)
YP_001483589	hypothetical (54)	YP_001484351	hypothetical (53)
YP_001483592	hypothetical (31)	YP_001484356	hypothetical (79)
YP_001483638	hypothetical (84)	YP_001484371	hypothetical (73)
YP_001483652	hypothetical (59)	YP_001484409	hypothetical (37)
YP_001483659	hypothetical (294)	YP_001484454[1,2]	hypothetical (38)
YP_001483660	hypothetical (270)	YP_001484479	hypothetical (104)
YP_001483662	hypothetical (237)	YP_001484485	hypothetical (49)
YP_001483663	hypothetical (343)	YP_001484490	hypothetical (106)
YP_001483793	possible Reverse transcriptase (84)	YP_001484508	hypothetical (54)
YP_001483823	hypothetical (115)	YP_001484513	hypothetical (48)
YP_001483910	hypothetical (75)	YP_001484515	hypothetical (48)
YP_001483934	hypothetical (130)	YP_001484518	hypothetical (91)
YP_001483943	hypothetical (97)	YP_001484542	hypothetical (52)
YP_001483945	hypothetical (64)	YP_001484701	hypothetical (43)
YP_001483978	hypothetical (37)	YP_001484803	hypothetical (38)
YP_001483980	hypothetical (42)	YP_001484807	hypothetical (60)
YP_001483986	hypothetical (39)	YP_001484831	hypothetical (38)
YP_001483993	hypothetical (47)	YP_001484833	hypothetical (50)
YP_001484001	hypothetical (64)	YP_001484848	hypothetical (112)
YP_001484007	hypothetical (43)	YP_001484850	hypothetical (38)
YP_001484015	hypothetical (100)	YP_001484851	hypothetical (54)
YP_001484025	hypothetical (57)	YP_001484868	hypothetical (39)
YP_001484029	hypothetical (42)	YP_001484876	hypothetical (64)
YP_001484108	hypothetical (49)	YP_001484879	hypothetical (98)
YP_001484113	hypothetical (57)	YP_001484902	hypothetical (75)
YP_001484132	hypothetical (41)	YP_001484961	hypothetical (51)
YP_001484175	hypothetical (44)	YP_00148496[6]	hypothetical (55)
YP_001484220	hypothetical (37)	YP_001484988[1]	hypothetical (130)
YP_001484237	hypothetical (50)	YP_001485055	hypothetical (34)

The low B/A ecotype Proclococcus clade include the following isolates: Pro. *marinus str. AS9601, Pro. marinus str. CCMP1986, Pro. marinus str. MIT9215, Pro. marinus str. MIT9301, Pro. marinus str. MIT 9312* and *Pro. marinus str. MIT 9515*. Most of these proteins are also found in *Pro. marinus str. MIT9202*, indicating that this strain also belongs to this subgroup.
